# Large Multichannel Architectures in Three-Dimensional Covalent Organic Frameworks for Efficient Guest Diffusion

**DOI:** 10.1021/jacs.6c01500

**Published:** 2026-04-13

**Authors:** Chenxi Xiong, Hui Zhou, Jiaming Zhou, Artit Jarusarunchai, Nuoqian Yan, Yoonseob Kim, Kwanwoo Nam, Dong-Myeong Shin, Seungkyu Lee

**Affiliations:** † Department of Chemistry, 25809 The University of Hong Kong, Pokfulam, Hong Kong SAR, China; ‡ Department of Mechanical Engineering, 25809 The University of Hong Kong, Pokfulam, Hong Kong SAR, China; ⊥ Department of Chemical and Biological Engineering, 121835 The Hong Kong University of Science and Technology, Hong Kong SAR, China; ¶ Department of Chemical Engineering and Materials Science, 26717 Ewha Womans University, Seoul 03760, South Korea

## Abstract

Guest diffusion in bulk covalent organic frameworks (COFs) is fundamentally limited by misaligned layers in two-dimensional (2D) frameworks and discontinuous 1D channels in 2D and 3D COFs across crystallite boundaries. Here, we report new triptycene-based 3D COFs, HKU-2-PEG*n* (HKU: The University of Hong Kong; *n* = 2 or 4), featuring orthogonally interconnected channels and tunable polyethylene glycol (PEG) functionality that mitigate the diffusion limitations. These COFs contain channels up to 4.8 nm in width and a total of 67 channels larger than 5 Å (13 crystallographically unique) distributed along multiple crystallographic directions, facilitating channel connectivity between randomly oriented crystallites. Lithium-ion (Li^+^) transport in bulk samples of these COFs exhibits high diffusion coefficients across a wide range of Li^+^ concentrations, comparable to those of commercial liquid electrolytes, demonstrating that large, multichannel crystalline architectures effectively mitigate the diffusion anisotropy inherent to low-dimensional channels in COFs.

## Graphical abstract

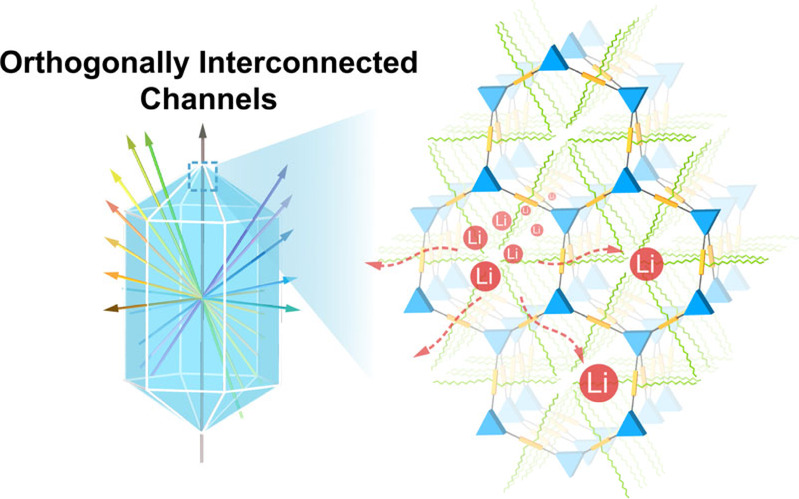

Covalent organic frameworks (COFs) are crystalline porous materials constructed from organic molecular units linked by strong covalent bonds.^1–3^ Reticular design enables precise control over connectivity, topology, and pore functionality in COFs, which is essential for diverse guest diffusion and interaction-related applications, such as gas storage and separation and energy storage.^4–6^

Guest diffusion in COFs is strongly influenced by lattice geometry, channel size, and functional groups installed.^7–11^ One-dimensional (1D) channels in 2D and 3D COFs have been widely studied for adsorption, separation, and catalysis and shown to support efficient diffusion.^12–18^ However, in principle, diffusion in the 1D channels of layered COFs can be hindered by layer misalignment.^19–21^ In bulk samples, diffusion through 1D channels of 2D and 3D COFs can be restricted by random crystal orientation.^22,23^ In contrast, 3D interconnected large channels have the potential to provide multidirectional pathways that reduce anisotropy, shorten detours around obstacles, and promote faster diffusion across diverse guest-migration scenarios.^22^

Bulky and flexible functional groups within COF channels can substantially alter the local dielectric environment, thereby modulating guest diffusion and solvation.^24–27^ Recent studies have demonstrated that incorporating such substituents—such as amine-bearing polymeric aliphatic chains for direct air capture and long polyethylene glycol chains for ion solvation—can induce selective diffusion and solvation in COFs.^28–32^ Despite this promise, COFs that integrate bulky functional groups within 3D interconnected large channels remain rare.^22,33^ The construction of 3D COFs with multiple large channels is inherently challenging, and the introduction of bulky, flexible linkers further complicates synthesis by disrupting lattice assembly and often yielding amorphous materials.^34–36^ Achieving large-pore 3D COFs that are readily functionalized would enable systematic investigations of mass transport across a wide range of guest-migration conditions.

Here, we report a new family of 3D COFs, HKU-2-PEG*n* (HKU: The University of Hong Kong; PEG: polyethylene glycol; *n* = 2 or 4), featuring 67 total channels (13 unique channels with apertures >5 Å) homogeneously and angularly distributed throughout a crystallite. With the largest pore aperture of 4.8 × 4.2 nm^2^, these channels are densely functionalized with long, flexible PEG chains, which provide desirable solvation environments for effective ion transport.^37^ The frameworks are in **acs**-topology and constructed by using a trigonal-prismatic node, 2,3,6,7,14,15-hexakis­(4-aminophenyl)­triptycene (HAPT), to induce a 3D topology, and a linear dialdehyde linker, 2,5-bis­(2-(2-methoxyethoxy)­ethoxy)­terephthalaldehyde (TAPEG*n*, *n* = 2 or 4), to increase node separation and open large channel apertures.^34^ The PEG chains occupy the channel volume, offering abundant interaction sites and promoting strong solvation with guest ions.

To evaluate the transport advantages of the designed architectures under realistic conditions, lithium-ion (Li^+^) migration was examined in bulk samples composed of randomly oriented crystallites. The materials exhibit room-temperature ionic conductivities of 0.74–1.20 mS cm^–1^ and high Li^+^ transference numbers of 0.62–0.65. The extracted Li^+^ diffusion coefficient reaches 1.6 × 10^–7^ cm^2^ s^–1^ at low Li^+^ concentrations, comparable to values reported for commercial liquid LiTFSI-propylene carbonate electrolytes. These experimental results indicate that the large multichannel architecture sustains intrinsic Li^+^ mobility despite random crystallite orientation.

Consistent with this observation, molecular dynamics simulations show that Li^+^ diffusion remains nearly isotropic even when pore overlap between neighboring crystallites is reduced to ∼50%, enabled by transport through the interconnected three-dimensional channel network.

HKU-2-PEG2 and -PEG4 were synthesized via a reversible imine condensation between HAPT and TAPEG2 and TAPEG4, respectively (Figure 1 and S1 to S14). In the synthesis condition, 2,2,2-trifluoroethylamine (TFEA) was used to control the crystallization kinetics, and trifluoroacetic acid (TFA) was used as a catalyst to improve the crystallinity.^38,39^ Single crystals with a trigonal morphology and an average size of ∼5 μm were obtained at the bottom of the reaction vials after heating at 40 °C for 7 days. Attempts to synthesize HKU-2 using a nonfunctionalized aldehyde linker instead produced an unknown phase crystal. The structure of HKU-2 could not be determined from the available PXRD data and is currently under further investigation (Figure S15).

**Figure 1:**
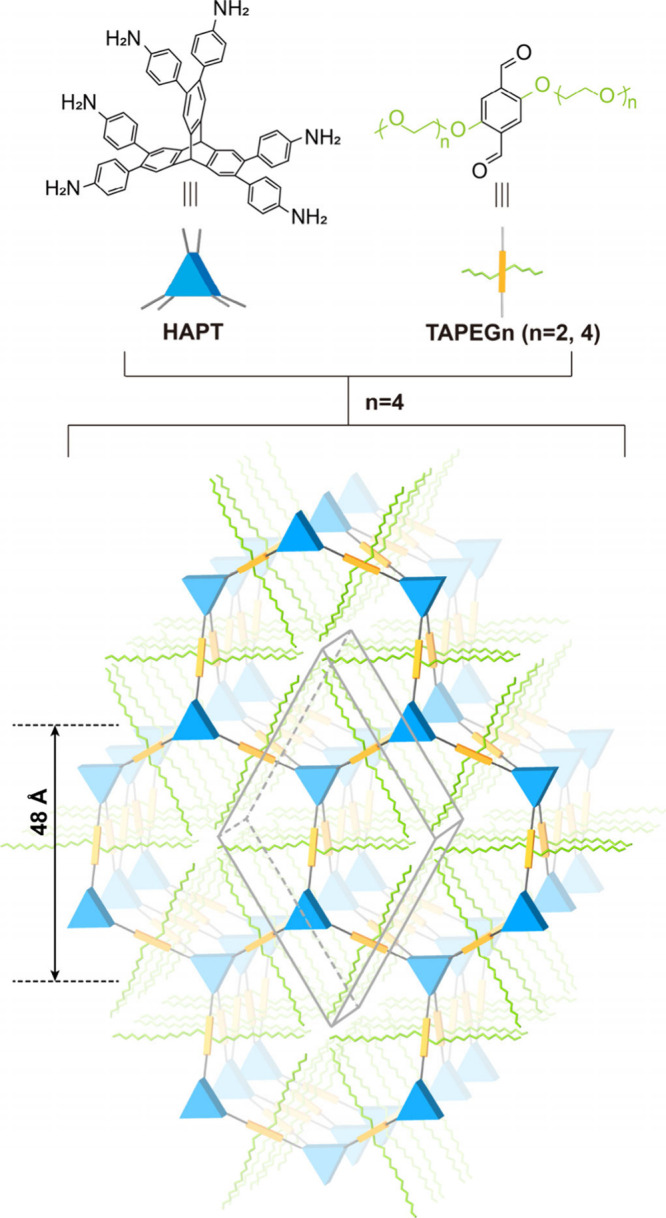
Structures of the building units and HKU-2-PEG*n* (*n* = 4). The blue trigonal prism represents HAPT, the linker is shown in gray, and the green chains represent the PEG units.

The structures of HKU-2-PEG2 and -PEG4 were validated by modeling their structures and comparing the simulated PXRD patterns with the experimentally obtained ones (Figure 2a and 2b). Both crystals were modeled in space group *P*6_3_/*m* and unit cell parameters, *a* = 41.60 Å and *c* = 22.80 Å for HKU-2-PEG2 and *a* = 41.93 Å and *c* = 26.20 Å for HKU-2-PEG4. The Pawley refinement suggested that the simulated patterns from the unit cell parameters are in good agreement with experimental patterns, with *R*_p_ and *R*_wp_, values of 3.63% and 5.27% for HKU-2-PEG4 and 1.39% and 1.81% for HKU-2-PEG4. Potential 2-fold interpenetrated versions of both COFs were modeled, and their simulated PXRD patterns were studied (Figure S16–S21 and Table S1). HKU-2-PEG4 presents larger unit cell parameters compared to HKU-2-PEG2, consistent with the significantly bulkier PEG chains occupying a larger pore volume. Molecular simulations indicate that the PEG chains preferentially adopt wall-associated conformations instead of fully spanning or blocking the channel interiors. As a result, while partial pore occupation by PEG is expected and indeed contributes to the solvation environment for Li^+^, continuous ion-transport pathways remain accessible. The Fourier-transform infrared (FT-IR) spectra of the COFs displayed a characteristic peak of imine (C=N) bonds at 1609 cm^–1^, confirming the successful imine bond formation (Figure 2c and S24). Additionally, the disappearance of N–H stretching bands (3300–3500 cm^–1^) from HAPT and a noticeable attenuation of the C=O stretching vibrations at 1678 cm^–1^ for TAPEG2 and 1683 cm^–1^ for TAPEG4 further support imine bond formation. SEM images (Figure 2d to g) revealed aggregated polyhedral particles, where HKU-2-PEG4 displayed higher uniformity than did HKU-2-PEG2. TEM images showed well-defined lattice fringes, confirming the high crystallinity of the COFs (Figure 2h and i). For HKU-2-PEG2, a lattice spacing of 36.0 Å was observed, corresponding to the (100) plane of the framework. In HKU-2-PEG4, the spacing slightly increases to 36.3 Å, indicating that the longer PEG chains induce a slight expansion.

**Figure 2:**
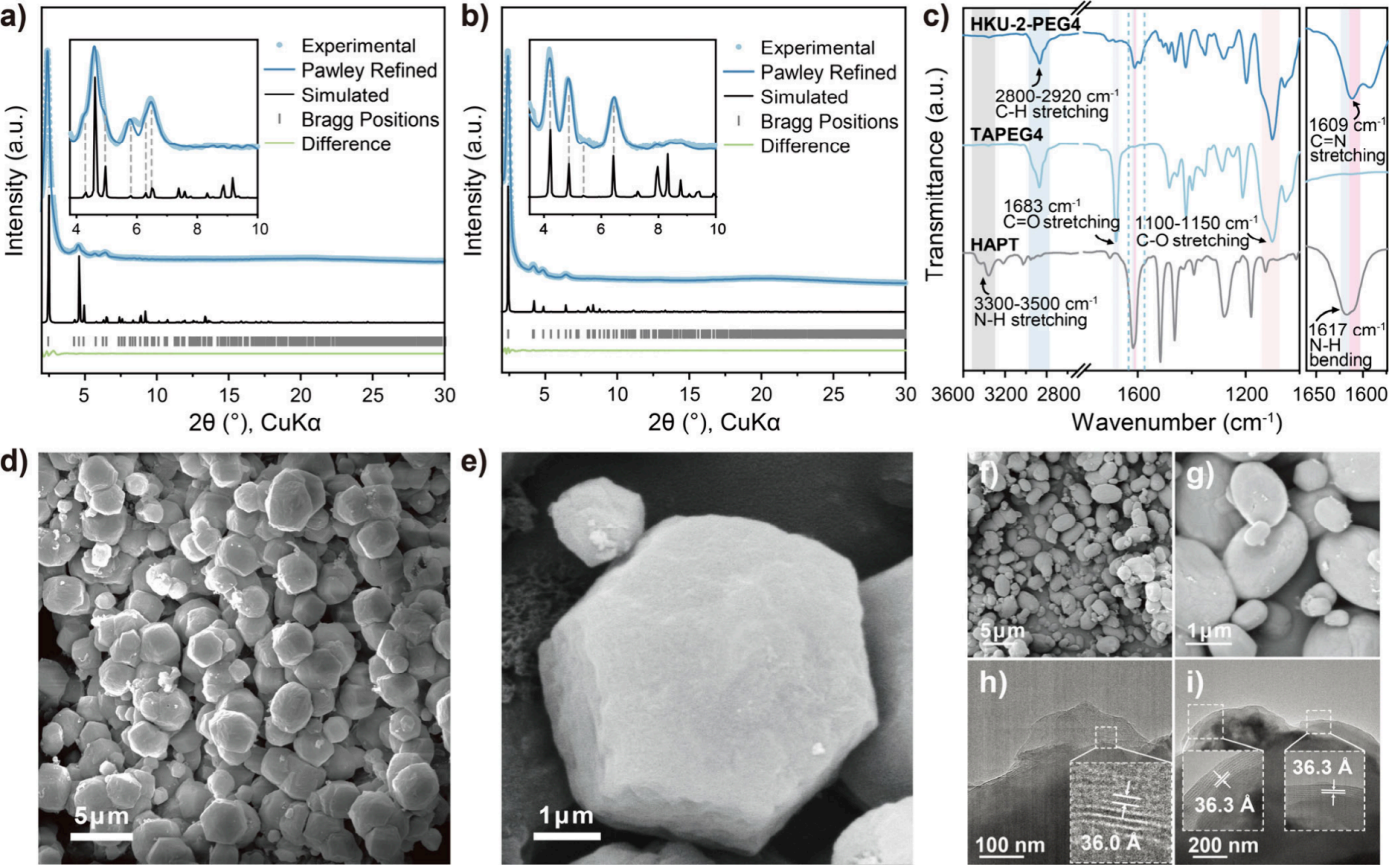
Structural and morphological analysis of HKU-2-PEG*n* (a) and (b) PXRD patterns of HKU-2-PEG2 and -PEG4, respectively. (c) FT-IR spectra of HKU-2-PEG2, TAPEG2, and HAPT. (d) and (e) SEM images of HKU-2-PEG4. (f) and (g) SEM images of HKU-2-PEG2. TEM images of (h) HKU-2-PEG2 and (i) HKU-2-PEG4.

The channel architecture of HKU-2-PEG4 was analyzed by rotating the crystal structure along multiple crystallographic axes (Figure 3 and Supporting Information Section 2.6). The orientations of these channels relative to the principal axis are illustrated in Figures 3a and 3b. Three distinct channel sets are present: one channel parallel to the principal axis, seven channels located along either the crystal edges or faces, and five channels occupying complementary positions. The channels are occupied by flexible and disordered PEG chains. Channel dimensions were measured between the arrowheads in Figure 3 without accounting for the van der Waals radii of the atoms. Eight unique channels with both width and height exceeding 10 Å were identified, providing sufficient space for the diffusion of small molecules and ions (Figure 3c to j). An additional five narrower channels (channel size between 5 and 10 Å) are shown in Figure 3k and 3o. By applying symmetry operations, including the 6_3_ screw axis, inversion center, and mirror plane, to the 13 crystallographically unique channels and excluding overlapping ones, a total of 67 channels were identified within a single crystal. These channels are well distributed angularly throughout the structure. Such an isotropic channel distribution increases the likelihood that when two crystallites come into contact with random orientations, even a small angular offset can bring their channels into alignment, thereby enabling intercrystallite channel connectivity.

**Figure 3:**
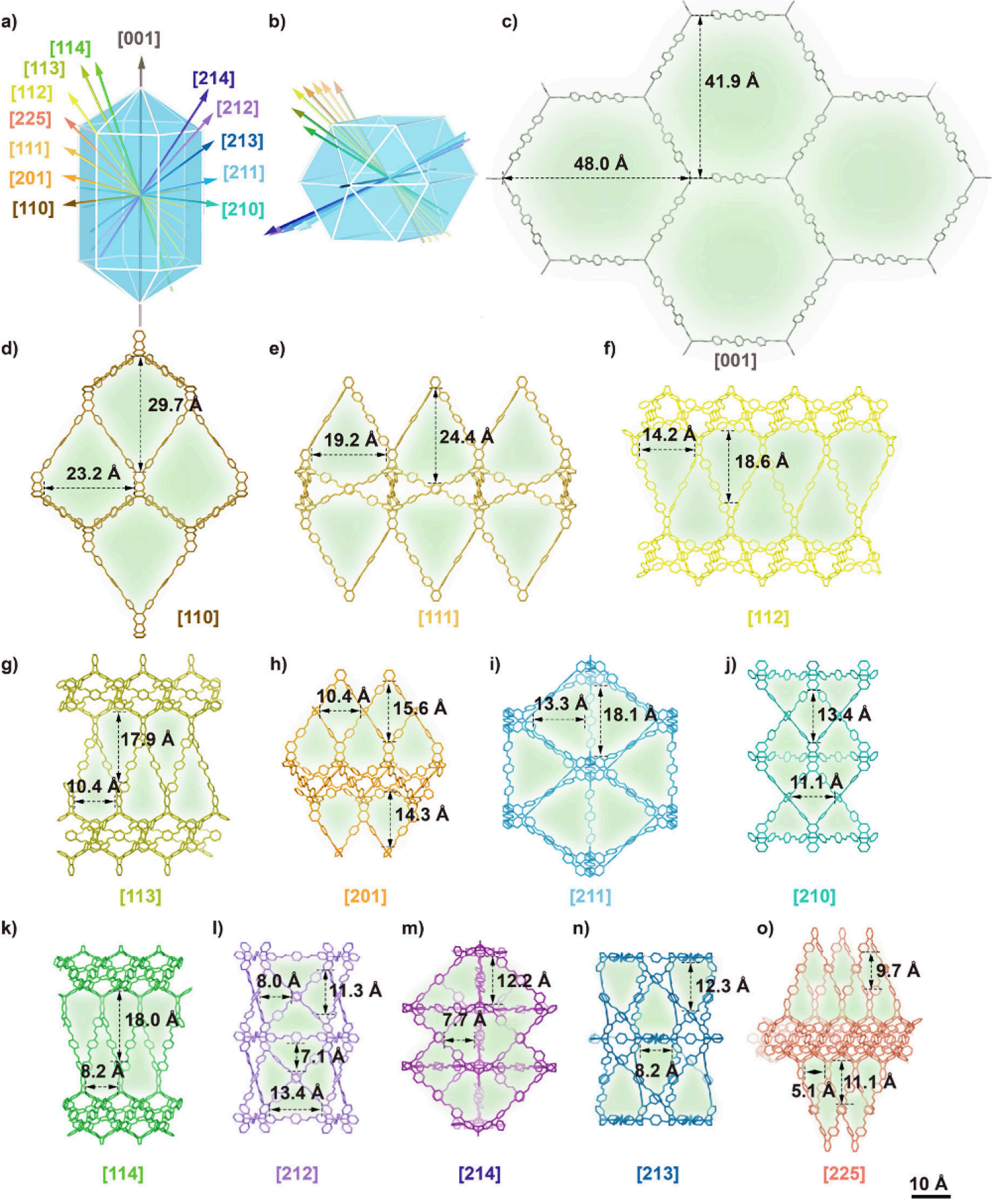
Channel structures and orientations in HKU-2-PEG4. (a and b) Relative orientations of the channels within a single crystal. Channel projections with both height and width greater than 10 Å (c to j) and between 5 Å and 10 Å (k to o). The frame colors correspond to the arrow colors in (a) and (b), indicating their respective directions. The labeled distances denote the separations between the arrow tips. The green pore regions represent disordered and flexible PEG chains occupying the channels.

The ionic conductivities and transference numbers (*t*_Li+_) of Li^+^-doped COFs soaked in propylene carbonate (PC) were examined using electrochemical impedance spectroscopy (Figure S35), which was then analyzed to determine the Li^+^ diffusion coefficients (*D*_*Li+*_) (Supporting Information Section S3.4). At room temperature (25 °C), Li^+^@HKU-2-PEG2 exhibits a high conductivity of 0.74 mS cm^–1^, while Li^+^@HKU-2-PEG4 reaches 1.20 mS cm^–1^ (Figure 4a). Li^+^@HKU-2-PEG2 delivered a *t*_Li+_ value of 0.62, while Li^+^@HKU-2-PEG4 achieved a similarly high value of 0.65 (Figure S36 and S37). For diffusion analysis, we investigated Li^+^ transport at varying Li^+^ salt concentrations, where the Li^+^ concentration is defined as the molar ratio of Li^+^ incorporated in the COF framework to the total amount of PC added to the electrolyte phase (Figure 4b). At low concentrations (∼2.8 M), Li^+^@HKU-2-PEG4 exhibits the *D*_*Li+*_ of 9.94 × 10^–8^ cm^2^ s^–1^, which is in excellent agreement with *D*_*Li+*_ in bulk PC solution,^40^ and this value decreases to 4.97 × 10^–9^ cm^2^ s^–1^ at elevated concentration (25.5 M). We attribute a lower diffusion coefficient at high concentration to ion pairing, in tandem with a change in solvation structure arising from the diminished PC/Li^+^ ratio.^41,42^ The activation energy calculated from the Arrhenius plot is 0.26 eV (Figure 4c), which is comparable to the bulk LiTFSI-PC system.^43^ To gain further insight into the mass transport in the COFs, the dominant diffusion mode of Li^+^ was assessed by comparing the characteristic diffusion length of Li^+^ (*L*_*c*_) to the radius of its solvation shell (*L*_*s*_). The characteristic length *L*_c_ can be quantified from the residence time of a solvation-shell member (τ_*res*_^*PC*^) and the *D*_*Li+*_ using the Einstein relation:^44^

$$L_{c}=\sqrt{6D_{Li+}{\tau_{res}}^{PC}}$$

where τ_*res*_^*PC*^ was extracted from extrapolations (Figure S41) of the experimental data reported by Pan et al.,^45^ and we set *L*_*s*_ = 6.6 Å in this study.^45,46^ Figure 4d displays the *L*_*c*_/*L*_*s*_ ratios obtained for each Li^+^ concentration. Across all concentrations examined, *L*_*c*_/*L*_*s*_ remains below unity, suggesting that diffusion occurred predominantly through solvation-shell reconfiguration rather than through solvation-cluster migration. This result is consistent with the experimental results^45^ and the molecular dynamics (MD) simulation^44^ of the liquid LiTFSI-PC system. Given low PC/Li^+^ ratios compared to the favorable coordination number (∼4 PC/Li^+^),^47,48^ the ether groups in PEG chains likely form clusters with Li^+^ in the process of diffusion throughout COFs. Enhanced Li^+^ mobility in Li^+^@ HKU-2-PEG4 (Figure 4b) corroborates enhanced ion solvation by ether groups arising from a greater number of coordination sites in longer PEG chains.

**Figure 4:**
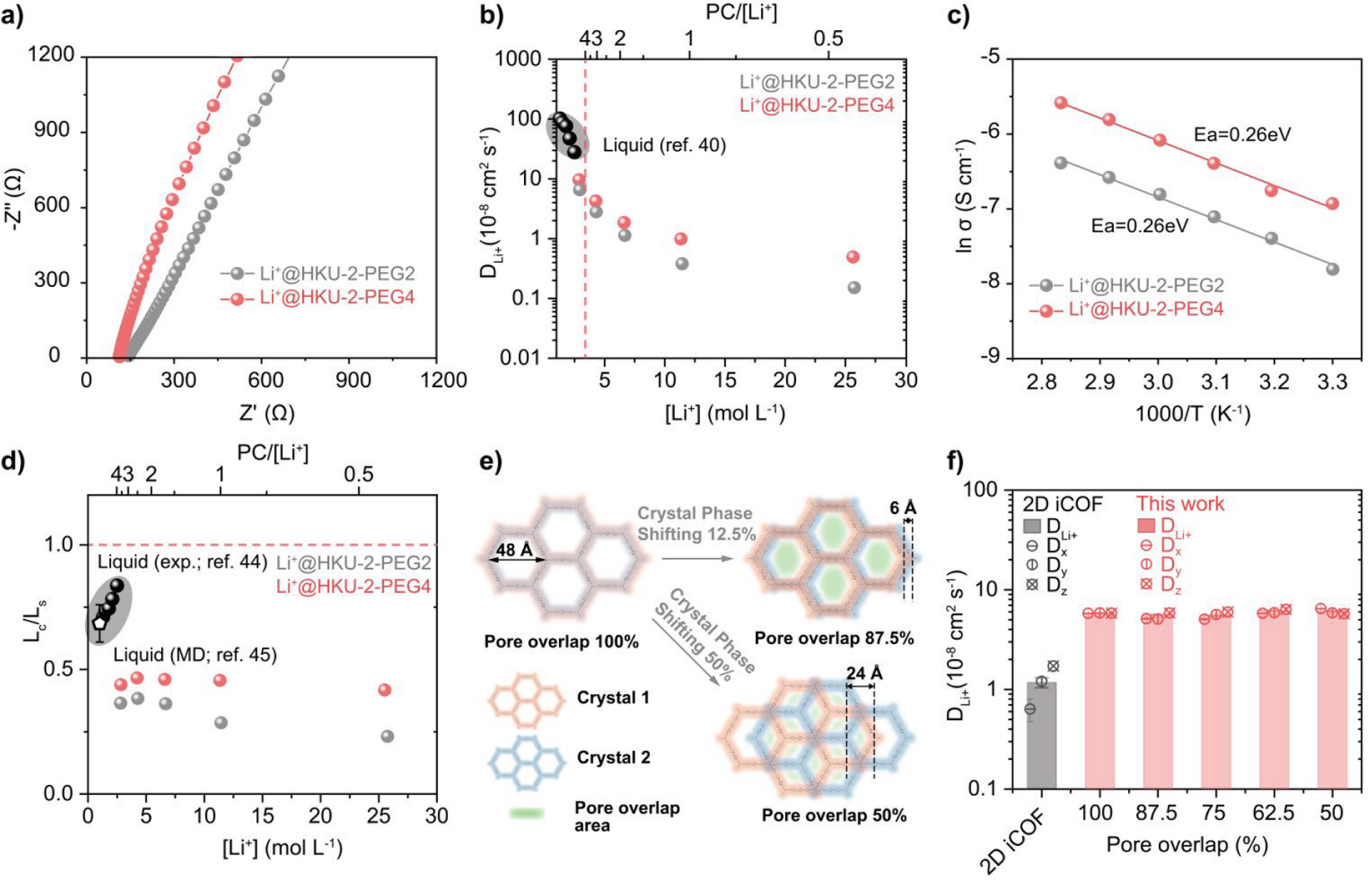
Diffusivity within Li^+^@HKU-2-PEG*n* (*n* = 2 or 4). (a) Nyquist plots of EIS measurements of Li^+^@HKU-2-PEG*n* COFs. (b) Diffusion coefficients for Li^+^ in Li^+^@HKU-2-PEG*n*. Black circles indicate Li^+^ diffusion coefficients at low concentration in PC solution.^40^ (c) Arrhenius plot of ionic conductivity as a function of temperature for Li^+^@HKU-2-PEG*n* COFs. (d) Ratio of the characteristic diffusion length (*L*_*c*_) to the solvation shell radius (*L_*s*_*). Black pentagon and circles are *L*_*c*_/*L*_*s*_ from MD simulations and experimental results at low concentration, respectively.^44,45^ (e) Schematic illustration of pore misalignment in Li^+^@HKU-2-PEG4. (f) Diagonal diffusion coefficients for Li^+^ in Li^+^@HKU-2-PEG4. Gray data points are simulation results of 2D iCOF.^49^

We performed MD simulations to elucidate the multidirectional Li^+^ transport pathways across the COFs. As illustrated in Figure 4e, two crystallites in contact were constructed with systematically varied degrees of interlayer pore misalignment. This misalignment was precisely tuned by shifting the HAPT moieties along the principal channel axis, corresponding to the largest pore dimension (48 Å). The four dislocations studied in this work are 6, 12, 18, and 24 Å, corresponding to pore overlaps spanning 87.5 to 50.0%. Figure 4f displays the diagonal diffusion coefficients in Li^+^-doped HKU-2-PEG4 as a function of differing pore overlaps. The *D*_*Li+*_ of COF with no misalignment was determined from MD simulations to be 5.82 × 10^–8^ cm^2^ s^–1^, agreeing with the experimentally measured value (4.26 × 10^–8^ cm^2^ s^–1^) at the given Li^+^ concentration. It is worth noting that isotropic diffusion was observed in HKU-2-PEG4, indicating that the multiple channels reduce anisotropy in mass transport, whereas directional diffusion was found to be dominant in the 2D ionic COF (iCOF).^49^ Additionally, visualizing the trajectories in Li^+^@HKU-2-PEG2 (see Movie S1 and Figure S42) reveals a tortuous path where Li^+^ ions interact with both immobile pore-confined PC molecules and skeletal ether groups, confirming the active role of PEG chains in coordinating Li^+^.

Even after pore misalignments were introduced, it was shown that the isotropic diffusion and its coefficient remained similar, suggesting that the interconnected channels shorten the detours around obstacles. We note that the 62.5% pore overlap slightly increases the diffusion coefficient (∼6%), largely due to the placement of ether moieties in close proximity, which improves ion solvation. Similar results were observed for HKU-2-PEG2 (Figure S40).

In summary, we have developed new 3D COFs, HKU-2-PEG*n* (*n* = 2 or 4), featuring well-defined, orthogonally interconnected channel networks that enable multidirectional Li^+^ transport in a crystallite. Li^+^ diffusion was quantitatively evaluated by diffusion coefficients derived from impedance measurements across a wide range of salt concentrations, revealing a strong dependence on the solvation structure and ion pairing. Analysis of characteristic diffusion lengths indicates that Li^+^ migration proceeds predominantly through solvation-shell reconfiguration, with PEG groups participating in Li^+^ coordination, particularly in frameworks bearing longer PEG chains. MD simulations further demonstrate that the interconnected 3D channel architecture supports largely isotropic Li^+^ diffusion and effectively mitigates the impact of pore misalignment. This work highlights the importance of large multiple channels in 3D COFs in enabling efficient guest diffusion.

## Supplementary Material





## Notes

Movie S1. Visualization of the trajectories in Li^+^@HKU-2-PEG2 (MP4)
Synthesis and characterization of the reported COFs, including TGA, FTIR, optical microscope images, and N_2_ isotherm measurements (PDF)
