# Supplementary material for: Large Multichannel Architectures in Three-Dimensional Covalent Organic Frameworks for Efficient Guest Diffusion
Source: J Am Chem Soc. 2026 Apr 13;148(15):15370–6. doi: 10.1021/jacs.6c01500 (PMC13107462; doi:10.1021/jacs.6c01500)
Supplement: Supplementary file 2 [file ja6c01500_si_002.pdf]

Supporting Information

Large multichannel architectures in three-dimensional covalent organic frameworks for efficient guest diffusion

Chenxi Xiong,[†,§] Hui Zhou,[‡,§] Jiaming Zhou,[‡] Artit Jarusarunchai,[†] Nuoqian Yan,[†] Yoonseob Kim,[⊥] Kwanwoo Nam,[¶] Dong-Myeong Shin,[‡,*] Seungkyu Lee[†,*]

[†]Department of Chemistry, The University of Hong Kong, Pokfulam, Hong Kong SAR, China

[‡]Department of Mechanical Engineering, The University of Hong Kong, Pokfulam, Hong Kong SAR, China

[⊥]Department of Chemical and Biological Engineering, The Hong Kong University of Science and Technology, Hong Kong SAR, China

[¶]Department of Chemical Engineering and Materials Science, Ewha Womans University, Seoul, 03760, South Korea

[*]Corresponding to dmshin@hku.hk and skchem@hku.hk

Table of contents

Section S1. Materials and instrumentation
Section S1.1. Materials
Section S1.2. Instrumentation
Section S1.3. Synthesis of starting materials
Section S1.4. Synthesis of HKU-2-PEG2 and HKU-2-PEG4
Section S1.5. Synthesis of Li^+@HKU-2-PEG2 and Li^+@HKU-2-PEG4
Section S2. Characterization of HKU-2-PEGn
Section S2.1. Powder X-ray diffraction (PXRD)
Section S2.2 Modelling and PXRD study of two-fold interpenetrated structure
Section S2.3. Unit cell parameters and fractional atomic coordinates
Section S2.4. Solid-state NMR spectra
Section S2.5. Fourier-transform infrared (FT-IR) spectra
Section S2.6. Channel characterization of HKU-2-PEGn
Section S2.7. Optical microscopy images
Section S2.8. N_2 isotherm measurements
Section S2.9. Thermogravimetric analysis (TGA)
Section S2.10. Transmission electron microscopy (TEM) images
Section S2.11. Stability test
Section S3. Conductivity studies
Section S3.1. Li^+ conductivity
Section S3.2. Li^+ transference number
Section S3.3. Electrochemical stability
Section S3.4. Calculation of Diffusion Coefficient.
Section S3.5. Molecular dynamic simulation
Section S3.6. Characterization of Li^+@HKU-2-PEGn
References
Figure S1-S50, Table S1-S5

Section S1. Materials and instrumentation

Section S1.1. Materials

Triptycene (purity ≥ 98 %), iron powder (purity ≥ 99 %), 4-(Boc-Amino)phenylboronic acid (purity ≥ 98 %), potassium carbonate (K_2CO_3, 99 %), tetrakis(triphenylphosphine)palladium ($Pd(PPh_3)_4$) (purity ≥ 99 %), sodium hydroxide (NaOH) (purity ≥ 98 %), were purchased from Energy Chemical Co. Ltd. 1,2-Dimethoxyethane was purchased from Macklin. Bromine (purity ≥ 99.8 %) was purchased form Acros Organics. Magnesium sulfate ($MgSO_4$, anhydrous, 99 %) was purchased from 3A Materials. Chloroform (99 %, GR), dichloromethane (99%, GR), methanol (99 %, GR), hexane (95%, GR), acetone (99.7%, HPLC), and acetic acid (99%, GR) were purchased from Duksan Pure Chemicals Co. Ltd. Hydrochloric acid (HCl, 36%, AR), dioxane (99%, AR), and tetrahydrofuran (THF, 99%, AR) were purchased from RCI Labscan Group Co. Ltd. All chemicals were used as received without further purification.

Section S1.2. Instrumentation

Powder X-ray Diffraction (PXRD) experiments were performed on Rigaku MiniFlex 600 Powder X-Ray Diffractometer using Cu metal target radiation source at 40 kV and 15 mA and D/teX Ultra2 detector. The activated sample was mounted on a zero-background sample holder and the diffraction pattern was scanned over the angular range of 2 to 45° (2θ) with a step size of 0.01 at room temperature. The optical images were recorded on a Leica M165 C microscope equipped with a flexacam C3 camera. Scanning electron microscope (SEM) images were recorded using a TESCAN MAIA3 XMH model high resolution Schottky FE-SEM. A beam intensity of 10 and an accelerating voltage of 10 kV were used to acquire the images. All samples were prepared on carbon tapes and coated with gold before SEM experiments. The Fourier-transform infrared (FT-IR) spectra were recorded on Spectrum two FT-IR spectrometer (PerkinElmer). The thermogravimetric analyses were conducted on TA Instrument Q50 TGA under N_2 atmosphere. The temperature range was set from 25 to 800 °C with a heating rate of 10 °C min^{-1}. Nuclear magnetic resonance (NMR) spectra were recorded on Bruker Ascend 500 NMR spectrometer at room temperature. Chemical shifts (*δ*) are reported in part per million (ppm) scale with respect to residual solvent signal as an internal reference. Multiplicities are reported as follows: s = singlet and d = doublet with corresponding coupling constants (*J*) in Hertz (Hz) and integration. The solid electrolyte pellet was prepared by Hydraulic Crimper (MTI MSK-160E). The Electrochemical impedance measurements,

transference number measurements and cyclic voltammetry measurements were conducted by Multichannel Potentiostat(Admiral Squidstat Plus).

Section S1.3. Synthesis of starting materials

Synthesis of 2, 3, 6, 7, 14, 15-hexabromotriptycene

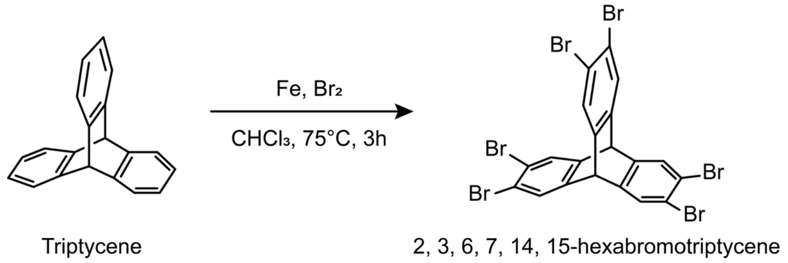

Figure S1. Synthesis of 2, 3, 6, 7, 14, 15-hexabromotriptycene.

2, 3, 6, 7, 14, 15-hexabromotriptycene was synthesized based on the reported literature[1] with slight modification. A solution of triptycene (2.00 g, 7.8 mmol) and iron powder (67.2 mg, 1.20 mmol) in chloroform (270 mL) was prepared in a reaction flask. A solution of bromine (2.55 mL, 49.7 mmol) in chloroform (70 mL) was added to the flask. The reaction mixture was heated under reflux at 75 °C for 3 h. Flash column chromatography was carried out before the solvent was removed under reduced pressure. The resultant crude product was triturated from dichloromethane and hexane. The precipitate was filtered and washed with methanol several times to give the desired 2, 3, 6, 7, 14, 15-hexabromotriptycene as a white solid: (4.95 g, 6.83 mmol, 88%); ^{1}H NMR (500 MHz, $CDCl_3$): δ (ppm) = 7.62 (s, 6 H), 5.24 (s, 2 H).

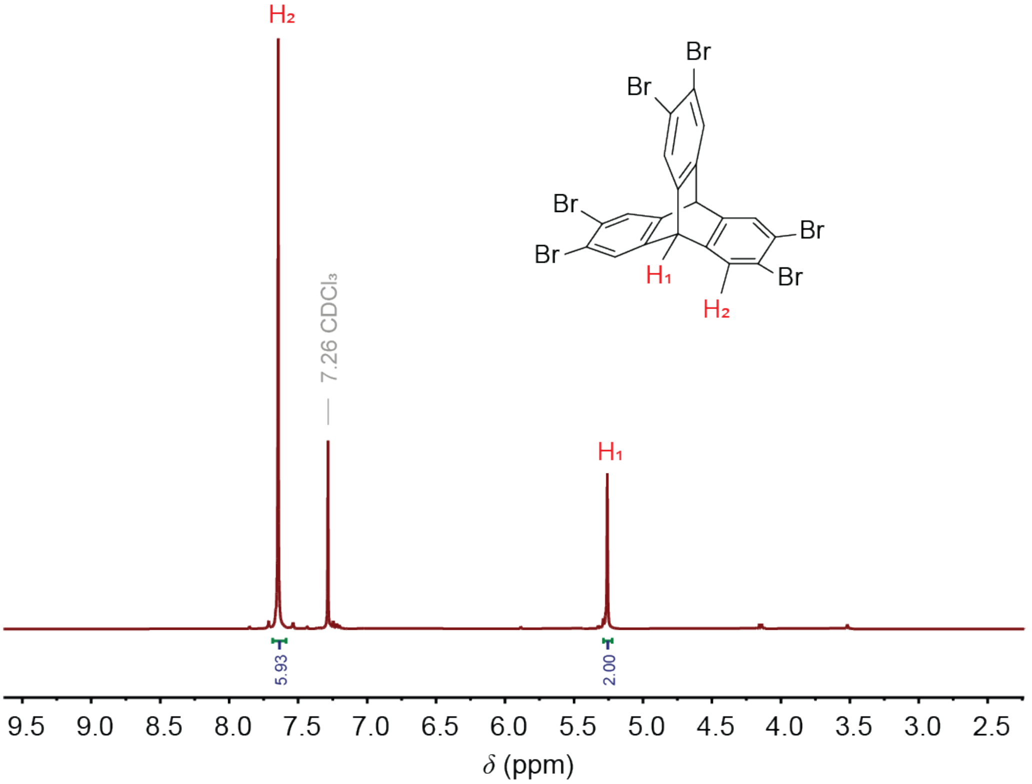

Figure S2. ^{1}H NMR Spectrum (500 MHz, $CDCl_3$, 298 K) of 2, 3, 6, 7, 14, 15-hexabromotriptycene.

Synthesis of HBocPT

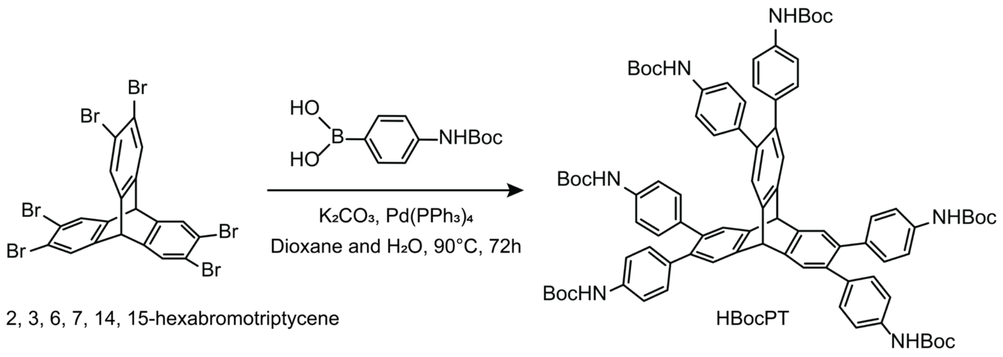

Figure S3. Synthesis of HBocPT.

A mixture of 2, 3, 6, 7, 14, 15-hexabromotriptycene (1.50 g, 2.06 mmol), 4-(Boc-Amino)phenylboronic acid (3.53 g, 15.88 mmol), K_2CO_3 (2.35 g, 16.98 mmol), and $Pd(PPh_3)_4$ (0.49 g, 0.55 mmol) was evacuated under vacuum, and backfilled with argon in the flask. Afterwards, a degassed mixture of dioxane (100 mL) and water (20 mL) was introduced into the flask. The reaction mixture was heated under reflux at 90 °C for 72 h. The reaction was

allowed to cool down to room temperature, and filtered through celite. After removing the solvent under reduced pressure, the crude product was dissolved in dichloromethane (50 mL). The solution was washed by extraction with water. The organic phase was combined and dried over anhydrous $MgSO_4$. After removing the solvent, the crude solid was purified by column chromatography (SiO_2, 2% EtOAc in CH_2Cl_2) to give the product as a colorless solid film (2.03 g, 70%); ^{1}H NMR (500 MHz, $(CD_3)_2SO$): δ (ppm) = 9.30 (s, 6H), 7.47 (s, 6H), 7.29 (d, J = 9.1 Hz, 12H), 6.92 (d, J=8.56 Hz, 12H), 5.87 (s, 2H), 1.45 (s, 54H). ^{13}C NMR (126 MHz, $(CD_3)_2SO$), δ (ppm) = 153.19, 144.22, 138.15, 136.95, 135.36, 130.03, 126.04, 117.92, 79.28, 51.69, 28.67.

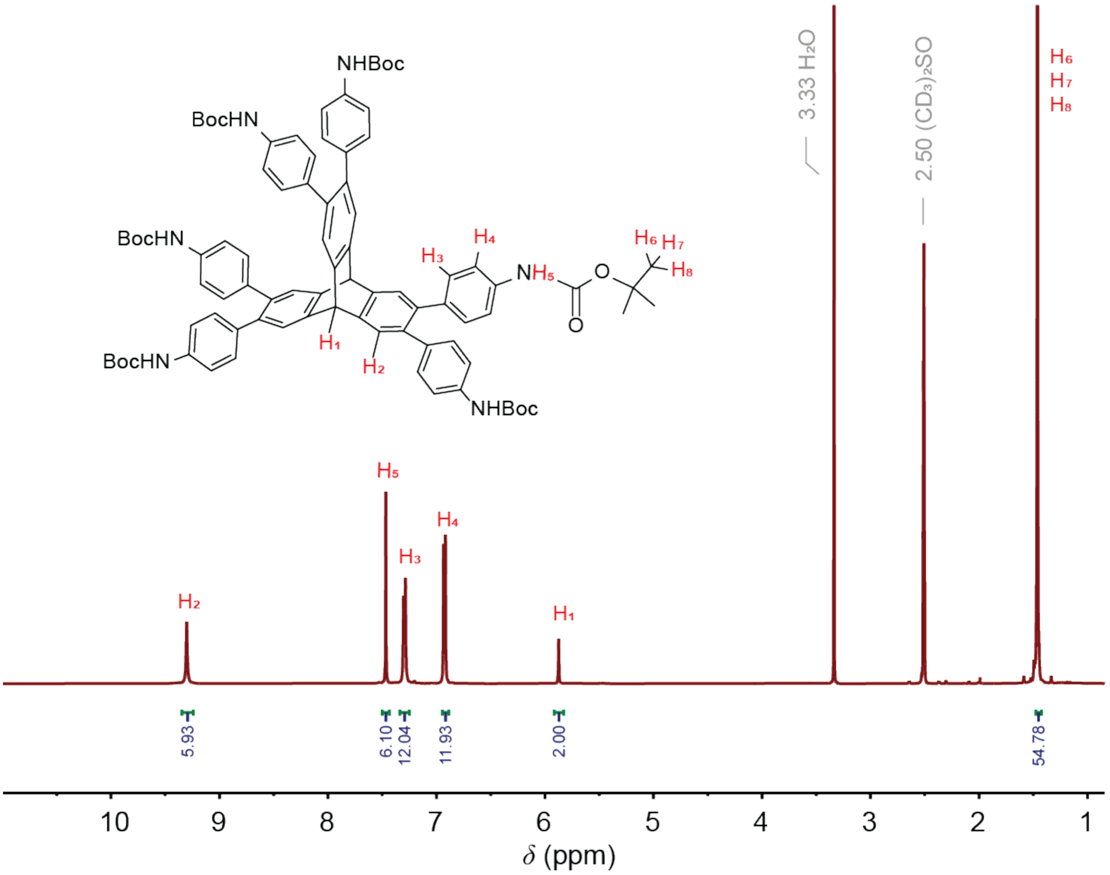

Figure S4. ^{1}H NMR Spectrum (500 MHz, $(CD_3)_2SO$, 298 K) of HBocPT.

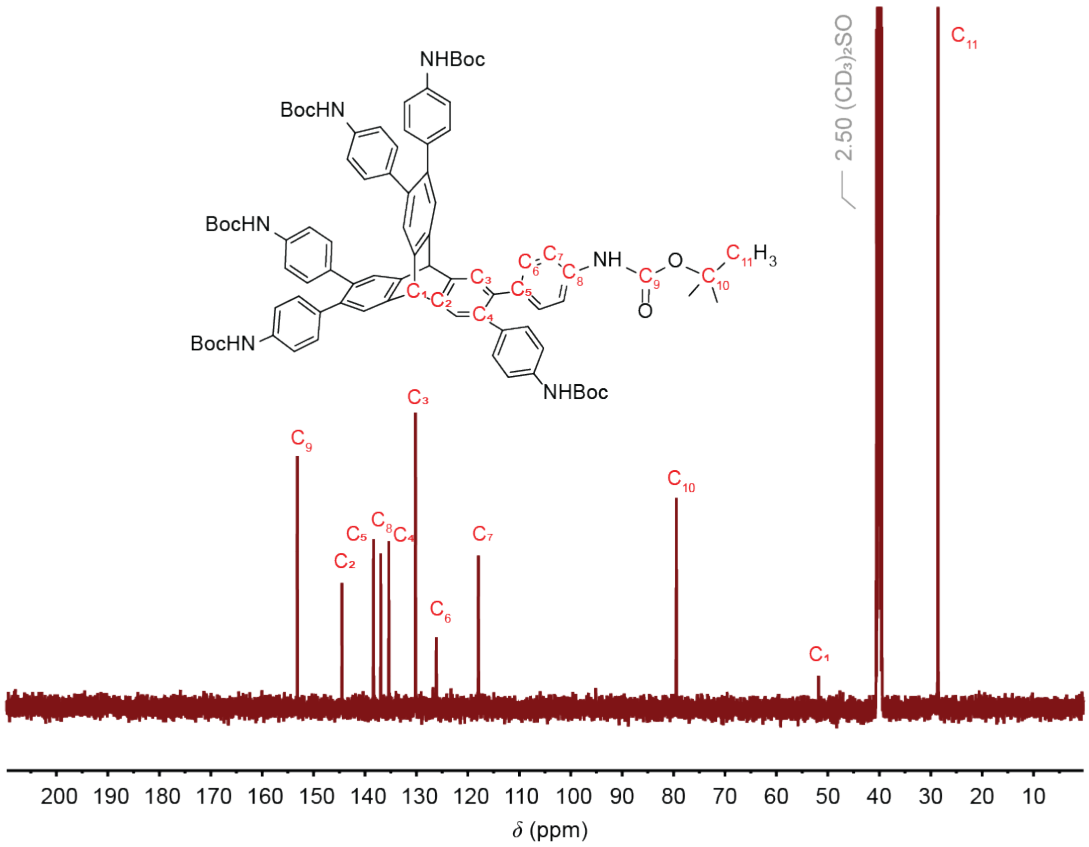

Figure S5. ^{13}C NMR Spectrum (126 MHz, $(CD_3)_2SO$, 298 K) of HBocPT.

Synthesis of HAPT

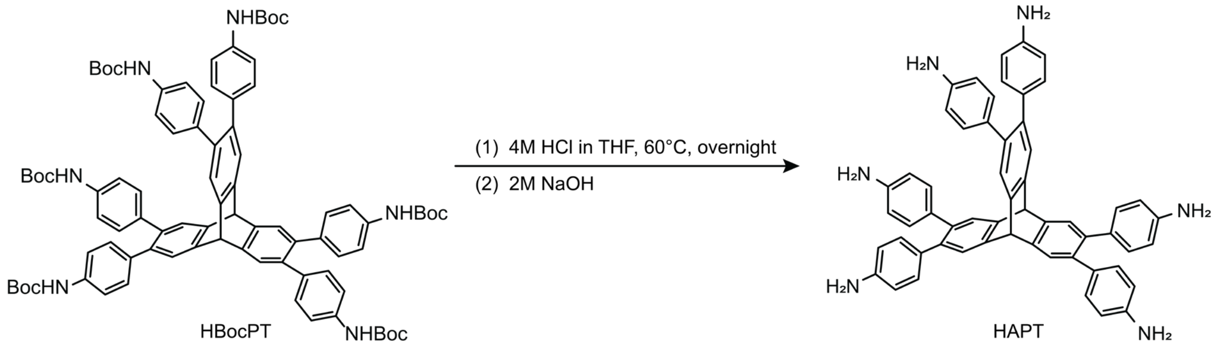

Figure S6. Synthesis of HAPT.

HBocPT (500mg, 0.36 mmol) was dissolved in THF (5 mL) in a 100 mL round-bottomed flask equipped with a magnetic stirrer bar. HCl in THF solution (4 M, 20 mL) was added and the resulting mixture was heated at 60 ˚C overnight. Upon cooling to room temperature, THF was removed under reduced pressure, and the remaining aqueous solution was basified with 2 M aqueous NaOH solution until pH = 9. The resulting white precipitate was collected by

centrifuge, washed with H_2O (200 mL) and dried under high vacuum to give the product as a white solid (262 mg, 92%). ^{1}H NMR (500 MHz, $(CD_3)_2SO$), δ (ppm) = 79.30 (s, 6H), 7.47 (s, 6H), 7.29 (d, J = 8.2 Hz, 12H), 6.93 (d, J = 8.4 Hz, 12H), 5.87 (s, 2H), 1.46 (s, 54H).. $_{13}$C NMR (126 MHz, $(CD_3)_2SO$), δ (ppm) = 148.25, 143.93, 137.17 130.25, 129.58, 125.59, 113.80, 51.97.

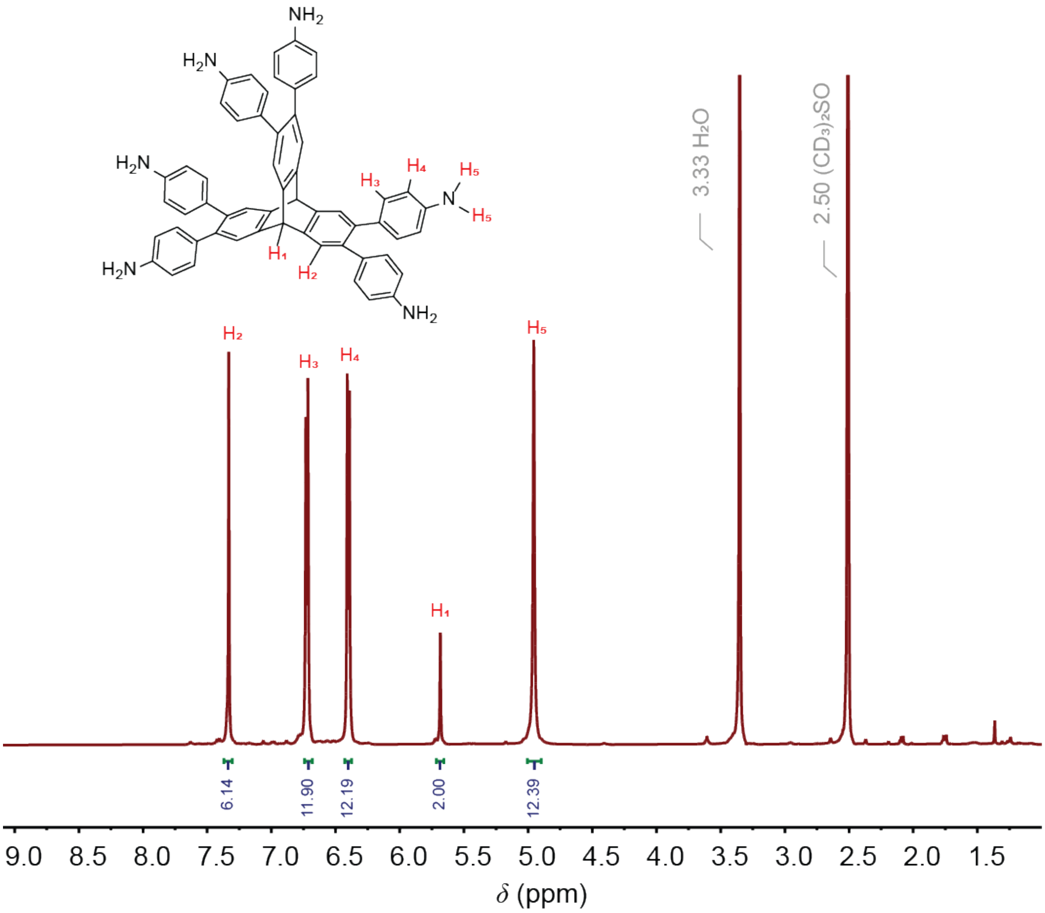

Figure S7. ^{1}H NMR Spectrum (500 MHz, $(CD_3)_2SO$, 298 K) of HAPT.

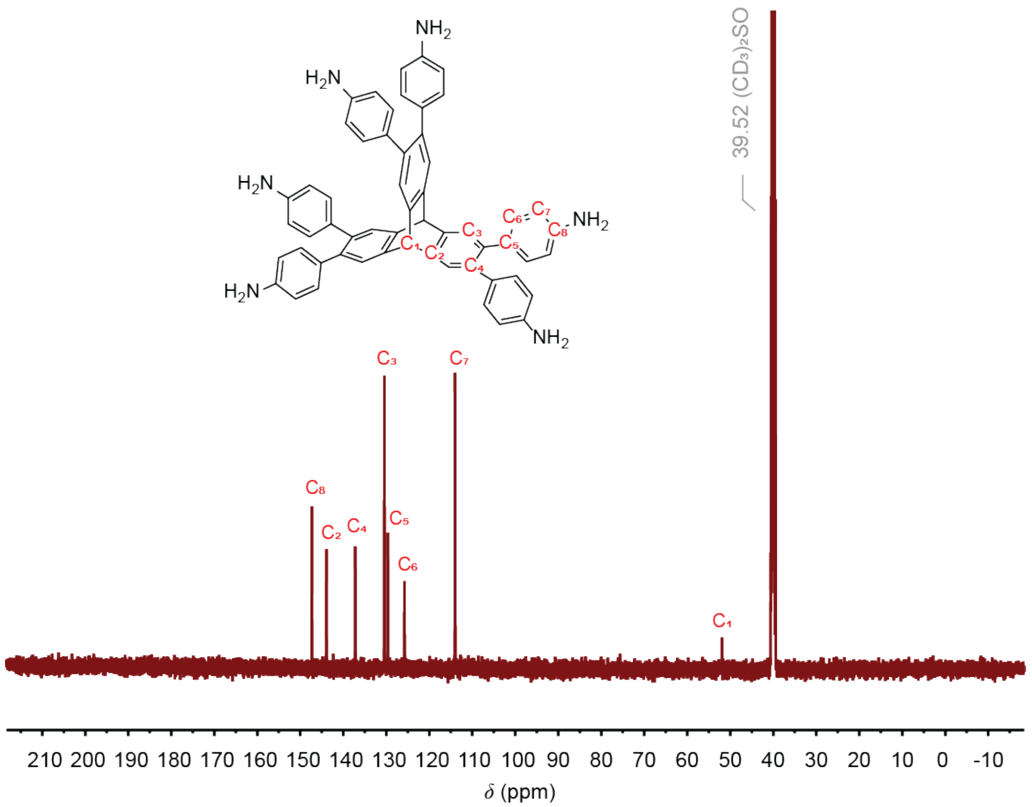

Figure S8. ^{13}C NMR Spectrum (126 MHz, $(CD_3)_2SO$, 298 K) of HAPT.

Synthesis of TAPEG2

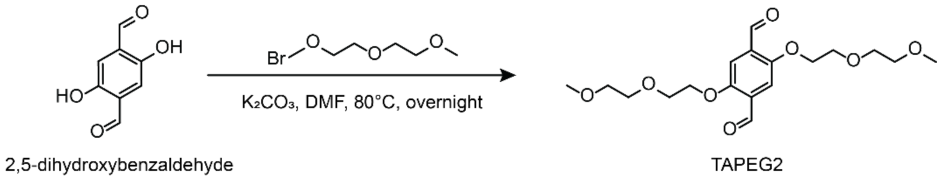

Figure S9. Synthesis of TAPEG2.

TAPEG2 was synthesized based on the reported literature. A solution of 2,5-dihydroxybenzaldehyde (276 mg, 2.00 mmol) and 1-bromo-2-(2-methoxyethoxy)ethane (797 µL, 6.00 mmol) in DMF (10 mL) was stirred at room temperature, followed by the addition of K_2CO_3 (1.66 g, 12.0 mmol). The reaction mixture was heated to 80 °C and stirred overnight. After cooling, the mixture was diluted with water (100 mL) and extracted with hot EtOAc (5 × 40 mL). The combined organic layers were dried over Na_2SO_4, filtered, and concentrated under reduced pressure. Purification by silica gel column chromatography (EtOAc) afforded the desired product as a pale yellow oil (286 mg, 0.835 mmol, 42%). ^1H NMR (500 MHz, $CDCl_3$), δ (ppm) = 10.55 (s, 2H), 7.48 (s, 2H), 4.32 – 4.29 (m, 4H), 3.94 – 3.91 (m, 4H), 3.74 – 3.71 (m, 4H), 3.61 – 3.58 (m, 4H), 3.41 (s, 6H).

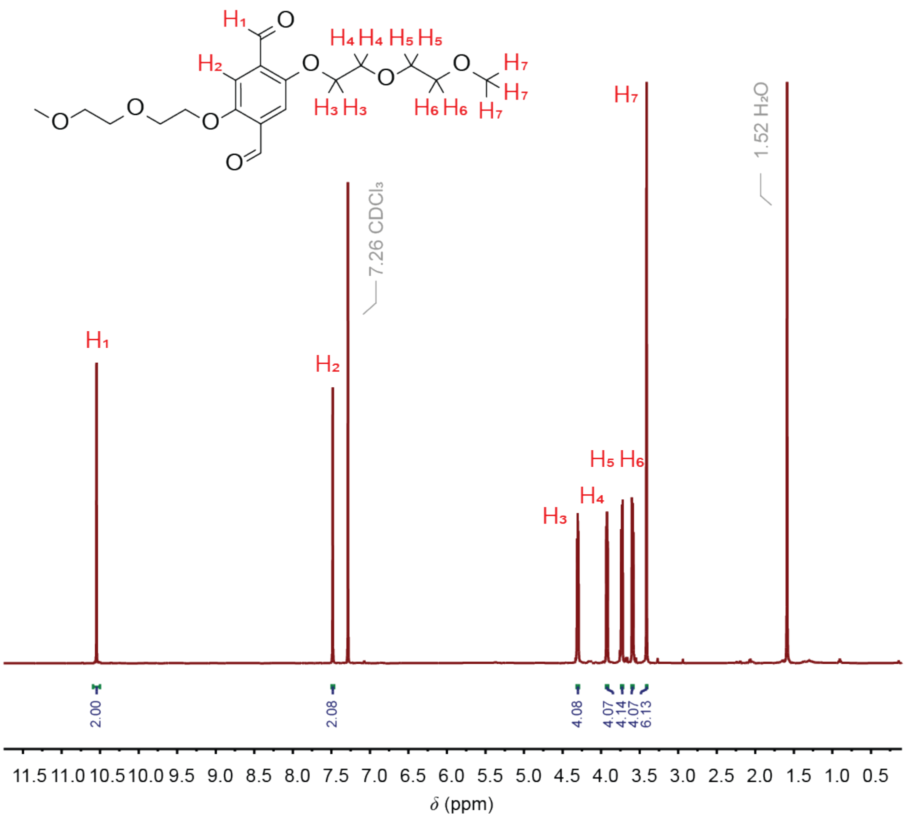

Figure S10. ^{1}H NMR Spectrum (500 MHz, $CDCl_3$, 298 K) of TAPEG2.

Synthesis of TAPEG4

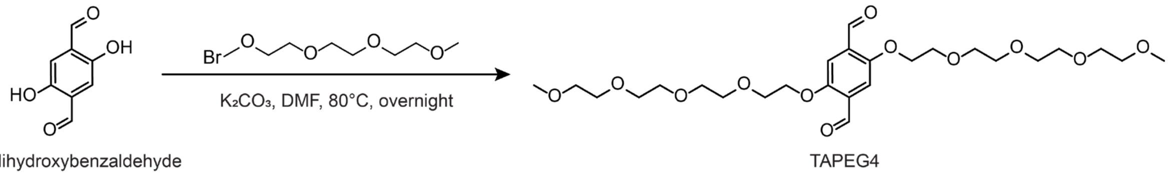

Figure S11. Synthesis of TAPEG4.

TAPEG4 was synthesized based on the reported literature[1] with slight modification. A solution of 2,5-dihydroxybenzaldehyde (276 mg, 2.00 mmol) and triethylene glycol 2-bromoethyl methyl ether (1.27 mL, 6.00 mmol) in DMF (10 mL) was stirred at room temperature, followed by the addition of K_2CO_3 (1.66 g, 12.0 mmol). The mixture was heated to 80 °C and stirred overnight. After cooling, the reaction was diluted with water (100 mL) and extracted with hot EtOAc (5 × 40 mL). The combined organic extracts were dried over Na_2SO_4, filtered, and concentrated under reduced pressure. Purification by silica gel column chromatography (EtOAc) afforded the desired compound as a pale yellow oil (502 mg, 0.971 mmol, 48%). ^{1}H NMR (500 MHz, $CDCl_3$), δ (ppm) = 10.54 (s, 2H), 7.47 (s, 2H), 4.31 – 4.27 (m, 4H), 3.94 –

3.90 (m, 4H), 3.73 (dd, J = 5.8, 3.2 Hz, 4H), 3.70 – 3.65 (m, 16H), 3.58 – 3.55 (m, 4H), 3.39 (s, 6H).

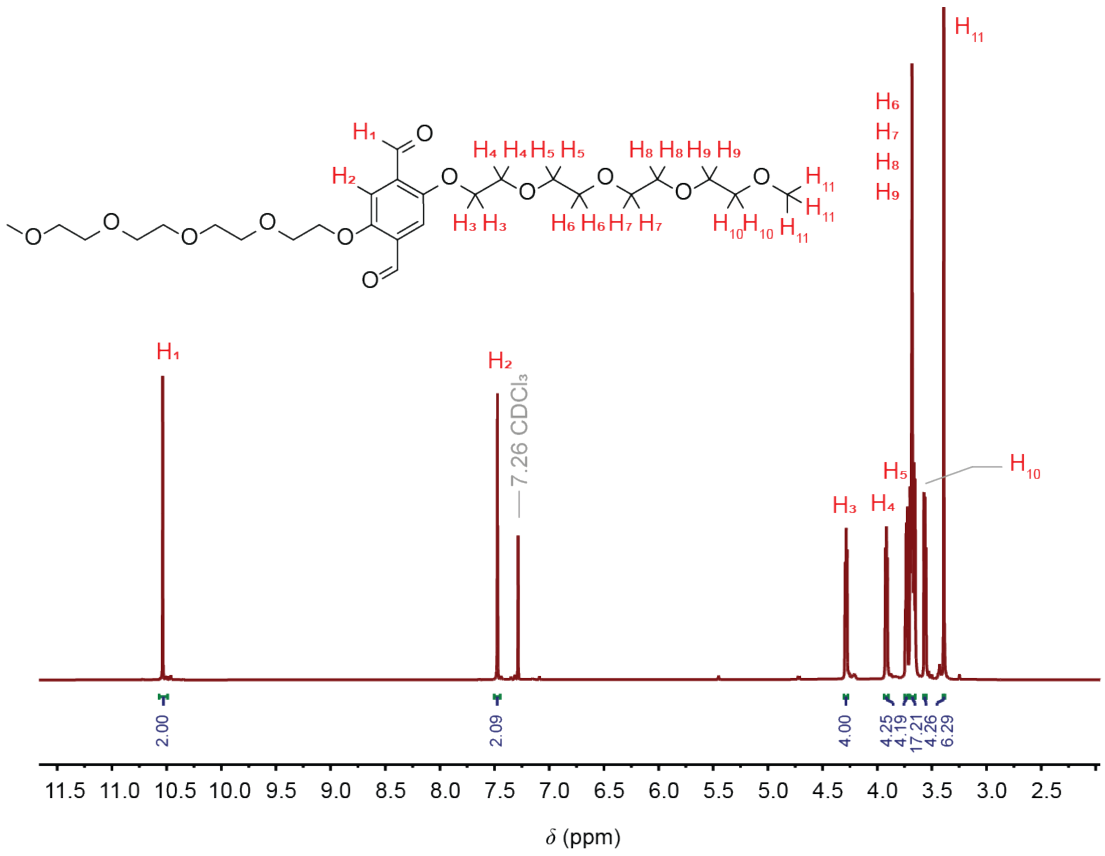

Figure S12. ^{1}H NMR Spectrum (500 MHz, $CDCl_3$, 298 K) of TAPEG4.

Section S1.4. Synthesis of HKU-2-PEG2 and HKU-2-PEG4

Synthesis of HKU-2-PEG2

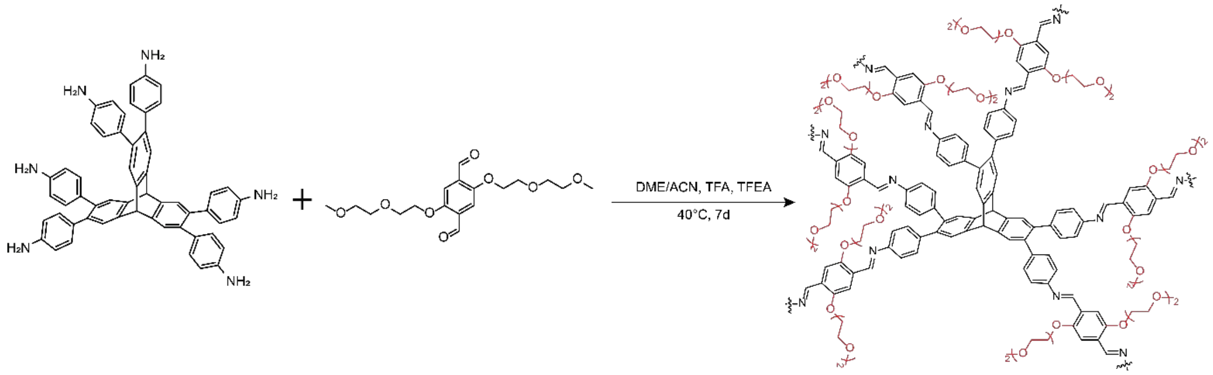

Figure S13. Synthesis of HKU-2-PEG2.

A scintillation vial (7 mL) with aluminum foil cap was added with TAPEG2 (11.11 mg, 0.03 mmol), DME (0.09mL) and ACN (0.01mL). TFEA (9.6 μL, 0.12 mmol) was added to the solution, followed by adding aqueous TFA (neat, 50 μL). HAPT (8 mg, 0.01 mmol), DME (0.36mL) and ACN (0.04mL) were added to the mixture. The resulting mixture was sonicated until red homogenous suspension was obtained. After sonication, the mixture was placed in a preheated oven at 40 °C and allowed to react undisturbedly for 7 days. The resulting HKU-2-PEG2 crystals were washed with DME (20 mL, 3 times per day for 3 days), followed by solvent-exchanged with anhydrous EtOH (20 mL, 3 times per day for 3 days). The EtOH-exchanged HKU-2-PEG2 crystals were transferred into a Kimwipe bag and activated via supercritical CO_2 to yield yellow crystals.

Synthesis of HKU-2-PEG4

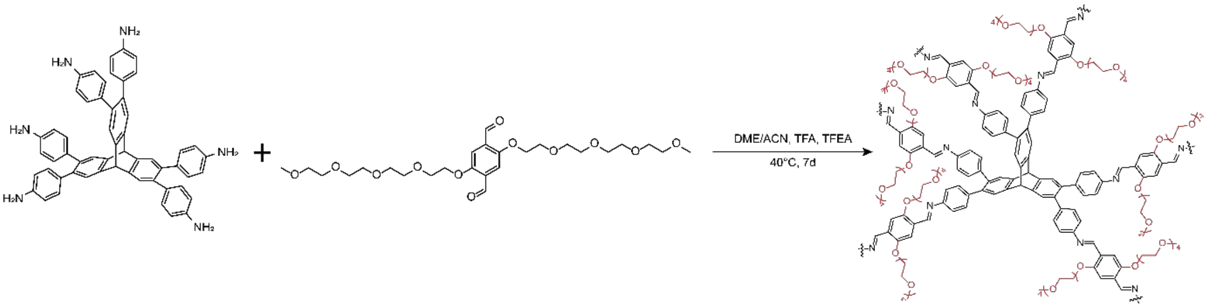

Figure S14. Synthesis of HKU-2-PEG4.

A scintillation vial (7 mL) with aluminum foil cap was added with TAPEG4 (16.40 mg, 0.03 mmol), DME (0.09mL) and ACN (0.01mL). TFEA (8 μL, 0.10 mmol) was added to the solution, followed by adding aqueous TFA (3M, 50 μL). HAPT (8 mg, 0.01 mmol), DME (0.36mL) and ACN (0.04mL) were added to the mixture. The resulting mixture was sonicated until red homogenous suspension was obtained. After sonication, the mixture was placed in a preheated oven at 40 °C and allowed to react undisturbedly for 7 days. The resulting HKU-2-PEG4 crystals were washed with DME (20 mL, 3 times per day for 3 days), followed by solvent-exchanged with anhydrous EtOH (20 mL, 3 times per day for 3 days). The EtOH-exchanged HKU-2-PEG4 crystals were transferred into a Kimwipe bag and activated via supercritical CO_2 to yield yellow crystals.

Section S1.5. Synthesis of Li+@HKU-2-PEG2 and Li+@HKU-2-PEG4

Synthesis of Li^+@HKU-2-PEG2 and Li^+@HKU-2-PEG4

HKU-2-PEGn (30 mg, n = 2 or 4) and a magnetic stir bar were placed in a 20 mL Schlenk flask. LiTFSI (120 mg) was dissolved in MeOH (3 mL) and added to the flask. The suspension was stirred overnight at room temperature, followed by vacuum stirring for 3 h. MeOH was then removed under vacuum at 120 °C for 5 h. The resulting solids were dried to yield Li^+@HKU-2-PEGn.

Section S2. Characterization of HKU-2-PEGn

Section S2.1. Powder X-ray diffraction (PXRD)

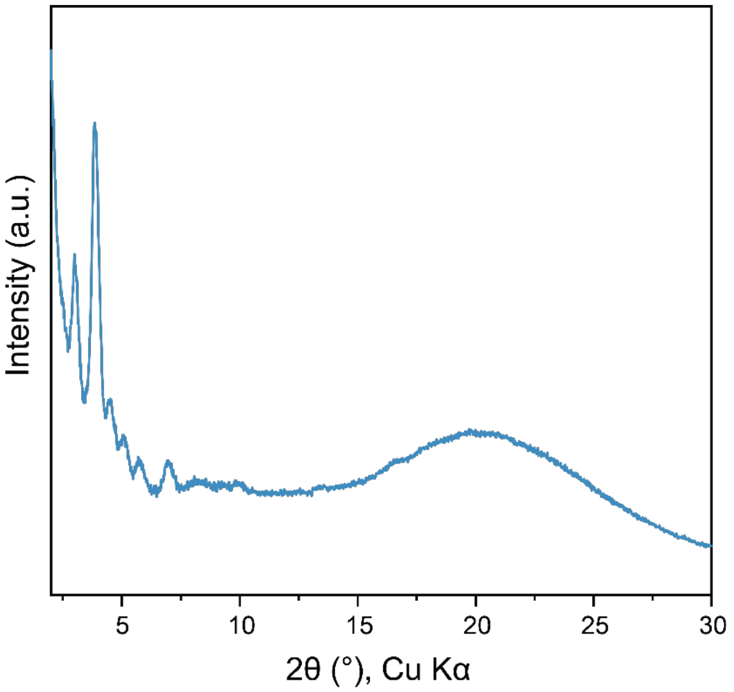

Figure S15. PXRD pattern of HKU-2 (unknown crystal).

Section S2.2. Modeling and PXRD study of two-fold interpenetrated structure

Possible two-fold interpenetrated structures of HKU-2-PEGn were constructed, and their simulated powder X-ray diffraction (PXRD) patterns were compared with the experimental data. For each material, two interpenetrated models were considered: one in which the second framework is located at the center of the pore (model 01 for HKU-2-PEG2, Figure S16; model 03 for HKU-2-PEG4, Figure S18) and the other in which the second framework is positioned close to the first framework (model 02 for HKU-2-PEG2, Figure S17; model 04 for HKU-2-PEG4, Figure S19).

For HKU-2-PEG2, the simulated PXRD pattern of model 01 shows a markedly different relative intensity between the (111) and (210) reflections compared with the experimental pattern, with the (111) reflection being significantly enhanced in the simulated data. In contrast, the simulated PXRD pattern of model 02 is broadly similar to the experimental pattern and the non-interpenetrated model, making it difficult to unambiguously distinguish this structural scenario based on PXRD alone.

For HKU-2-PEG4, the simulated PXRD patterns of both interpenetrated models (model 03 and model 04) differ clearly from the experimental data. Additional reflections indexed to (11-1), (201), and (001) appeared in the simulated patterns of model 03 and model 04 but are absent in experimental PXRD pattern. Moreover, model 03 exhibits a significantly weaker (200) reflection relative to the (101) reflection, which is inconsistent with the experimental intensity distribution. Overall, the experimental PXRD patterns of HKU-2-PEG4 are inconsistent with the two-fold interpenetrated models considered.

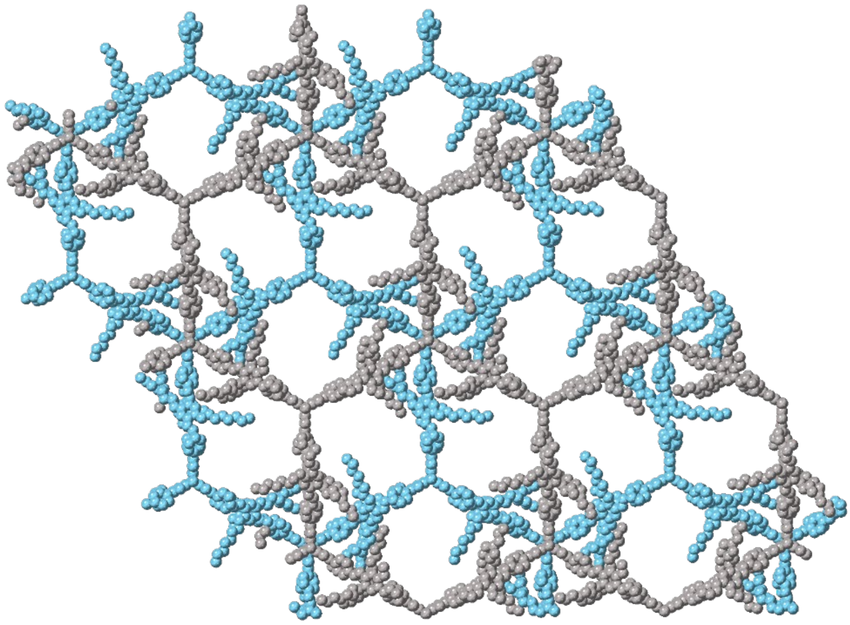

Figure S16. Model (model 01) of two-fold interpenetrated HKU-2-PEG2.

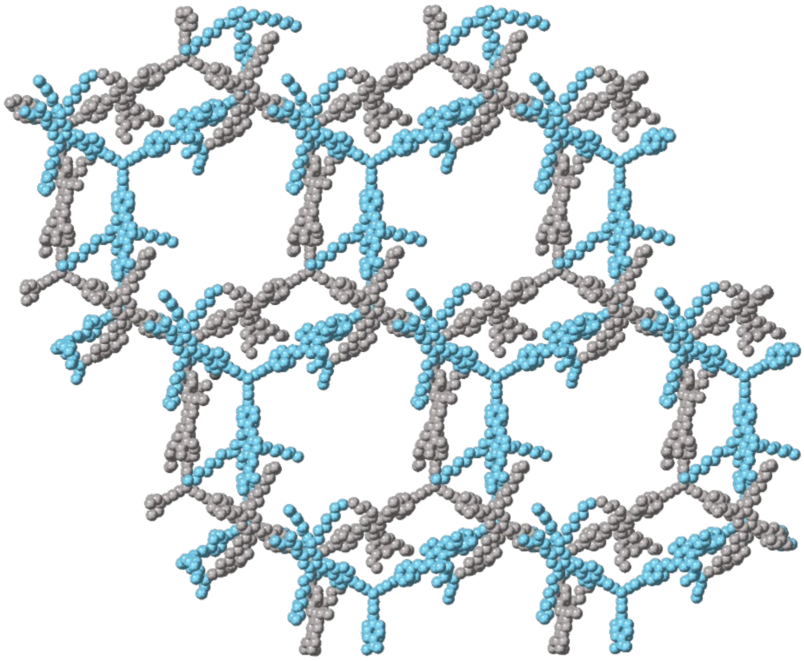

Figure S17. Model (model 02) of two-fold interpenetrated HKU-2-PEG2.

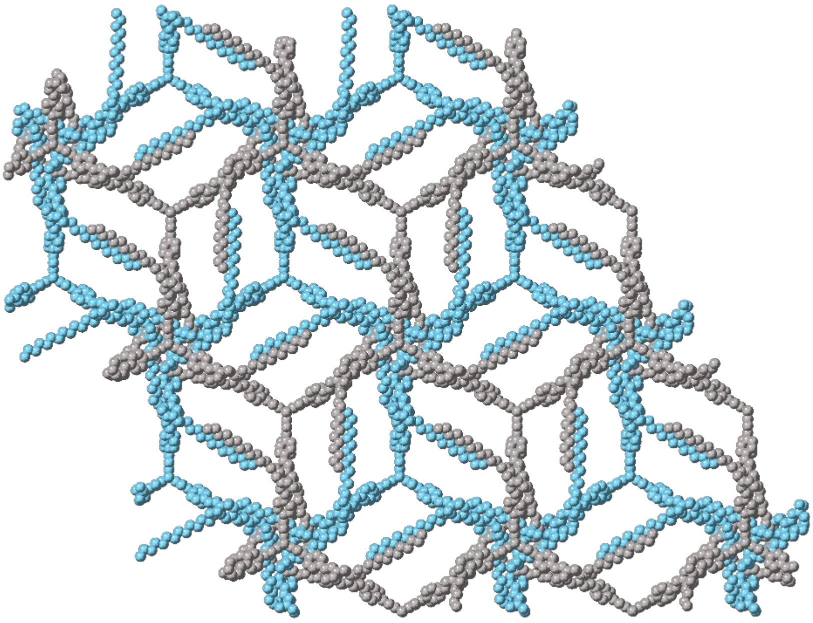

Figure S18. Model (model 03) of two-fold interpenetrated HKU-2-PEG4.

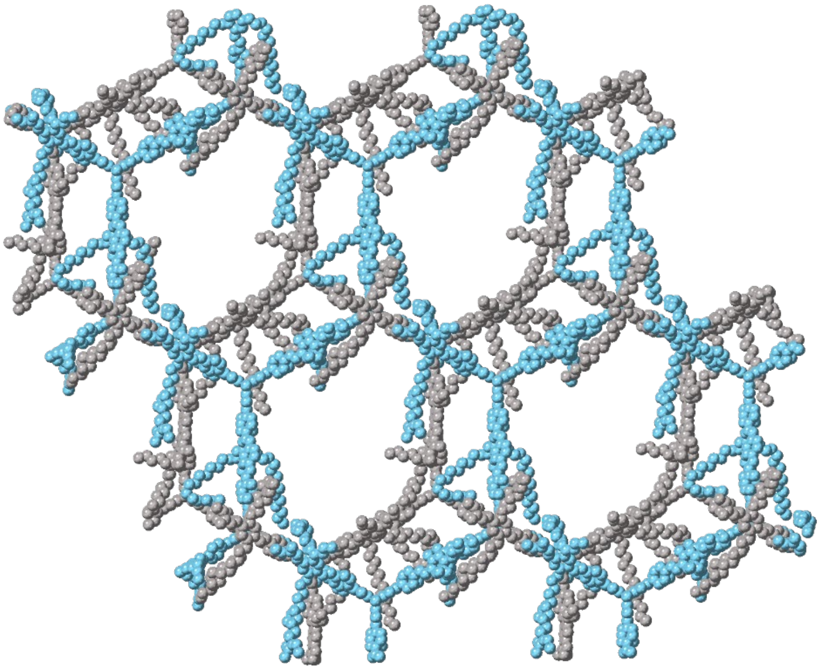

Figure S19. Model (model 04) of two-fold interpenetrated HKU-2-PEG4.

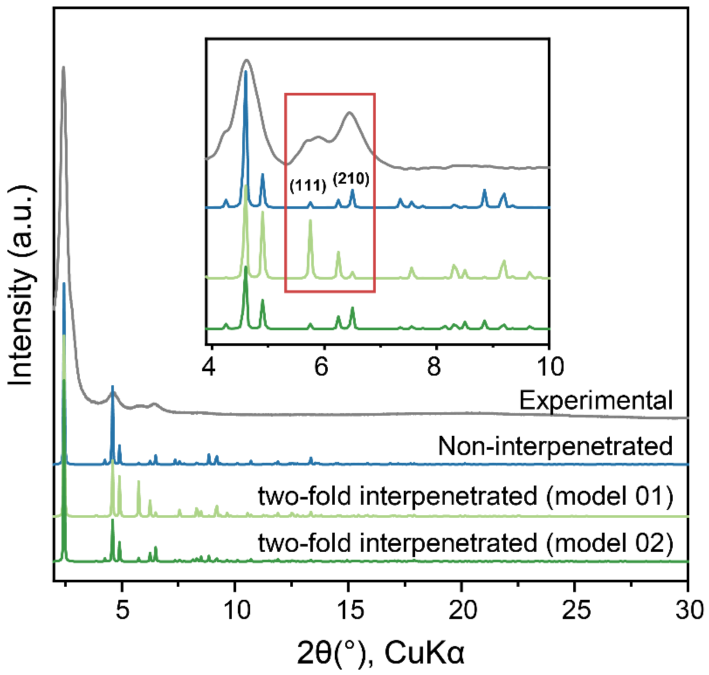

Figure S20. Comparison of PXRD patterns for HKU-2-PEG2: experimental (grey), non-interpenetrated (dark blue), two-fold interpenetrated model 01 (pale green), two-fold interpenetrated model 02 (dark green).

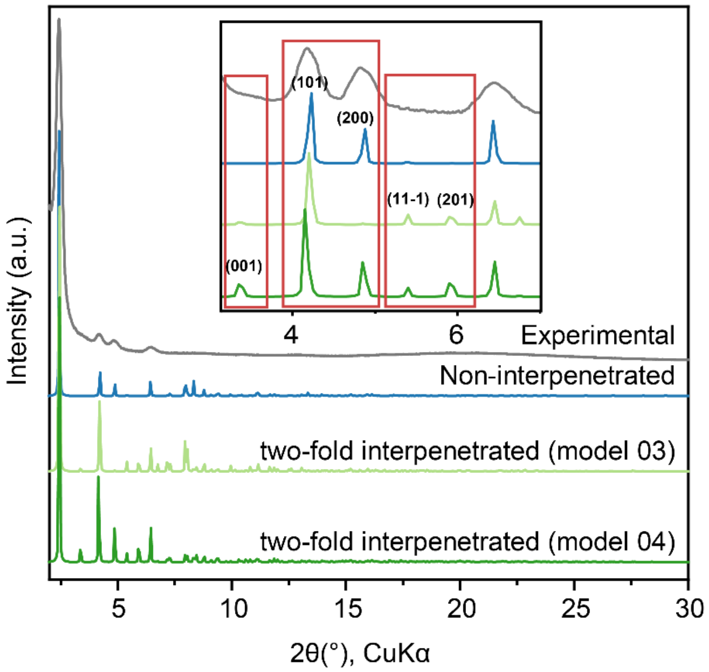

Figure S21. Comparison of PXRD patterns for HKU-2-PEG4: experimental (grey), non-interpenetrated (dark blue), two-fold interpenetrated model 03 (pale green), two-fold interpenetrated model 04 (dark green).

Geometry Optimization simulations were conducted using the Forcite module in Materials Studio with the universal force field. After optimization and equilibration, the total energies were obtained (Table S1). The two-fold interpenetrated models contain a higher framework density and a larger number of atoms per unit cell compared to the non-interpenetrated structures. Accordingly, the total energies reported in Table S1 correspond to fully optimized periodic models and were intended for qualitative comparison of structural trends rather than strict thermodynamic evaluation normalized per formula unit.

Table S1. Comparison of calculated thermodynamics energy for non-interpenetrated and two-fold interpenetrated of HKU-2-PEG2 and HKU-2-PEG4 at 295K.

	Total Energy (kcal/mol)		Total Energy (kcal/mol)
HKU-2-PEG2 non-interpenetrated	855.042	HKU-2-PEG2 non-interpenetrated	1210.492
HKU-2-PEG2 two-fold interpenetrated (model 01)	1516.726	HKU-2-PEG4 two-fold interpenetrated (model 03)	2144.709
HKU-2-PEG2 two-fold interpenetrated (model 02)	1392.257	HKU-2-PEG4 two -fold interpenetrated (model 04)	2094.286

Section S2.3. Unit cell parameters and fractional atomic coordinates

Table S2. Unit cell parameters and fractional atomic coordinates for HKU-2-PEG2.

Space group		$P6_3/m$	
Unit cell		a=b=41.6000 Å, c=22.8000 Å, α = γ = 90°, β=120°	
Atoms	x	y	z
C1	0.44034	0.60929	1.17619
C2	0.40185	0.593	1.18458
C3	0.39309	0.53965	1.12743

C4	0.45514	0.5909	1.14382
C5	0.43152	0.55569	1.11879
N6	0.44484	0.53516	1.08492
C7	0.64737	0.35297	0.71898
H8	0.45998	0.63775	1.19571
H9	0.37326	0.5112	1.10808
H10	0.48644	0.60439	1.13761
C11	0.62162	0.44194	0.6597
C12	0.48887	0.52176	0.46629
C13	0.52622	0.53582	0.48255
C14	0.5374	0.5146	0.51572
C15	0.73931	0.36906	1.81191
C16	0.77354	0.38593	1.78128
C17	1.47892	0.54593	1.43095
H18	0.65291	0.45538	0.66591
H19	0.54818	0.56503	0.4688
H20	0.73904	0.36876	1.86191
H21	1.50147	0.57484	1.41698
O22	0.57413	0.5299	0.53017
C23	0.40281	0.4837	0.44264
C24	0.36679	0.4639	0.44289
O25	0.34339	0.47194	0.40759
C26	0.30228	0.45097	0.41326
C27	0.28318	0.46878	0.37725
O28	0.24288	0.44088	0.37978
C29	0.22164	0.46014	0.36088
H30	0.41197	0.49058	0.39496
H31	0.40854	0.5106	0.46488
H32	0.35749	0.46262	0.49029
H33	0.35785	0.43424	0.42915
H34	0.29459	0.44996	0.46149
H35	0.29109	0.42141	0.39667

H36	0.29333	0.47348	0.3299
H37	0.28959	0.49621	0.39832
H38	0.19132	0.44256	0.37445
H39	0.22346	0.46376	0.31121
H40	0.23424	0.48851	0.38283
C41	0.33333	0.66667	0.19112

Table S3. Unit cell parameters and fractional atomic coordinates for HKU-2-PEG4.

Space group		*P*6₃/*m*	
Unit cell		a=b= 41.9305 Å, c=26.2000 Å, α = γ = 90°, β=120°	
Atoms	x	y	z
C1	0.44287	0.60443	1.184
C2	0.40452	0.59143	1.18935
C3	0.39518	0.54241	1.12727
C4	0.45698	0.58683	1.15117
C5	0.43331	0.55518	1.12177
N6	0.44572	0.53517	1.08721
C7	0.64652	0.35348	0.72314
O8	0.42694	0.46779	0.46422
C9	0.39817	0.45416	0.50161
C10	0.39766	0.48572	0.53008
O11	0.36774	0.47087	0.56447
C12	0.36481	0.49969	0.58765
C13	0.33475	0.48349	0.62826
O14	0.33194	0.51205	0.65277
C15	0.30058	0.51309	0.6355
C16	0.3706	0.62888	0.19652
C17	0.38853	0.61124	0.27782
C18	0.38124	0.5601	0.34043
C19	0.47929	0.54366	0.42816

C20	0.48879	0.5204	0.46515
C21	0.46317	0.4846	0.48292
C22	0.47454	0.46489	0.51649
C23	0.69921	0.45335	0.34186
O24	0.7327	0.45476	0.35392
C25	0.73213	0.42292	0.33507
C26	0.77083	0.42839	0.33799
O27	0.77097	0.39788	0.31552
C28	0.80666	0.40266	0.319
H29	0.46229	0.62891	1.20449
H30	0.37576	0.51843	1.1058
H31	0.48646	0.59823	1.14902
H32	0.40025	0.43504	0.5289
H33	0.3715	0.43814	0.48131
H34	0.39496	0.50388	0.50141
H35	0.42405	0.50171	0.55124
H36	0.35724	0.51424	0.55876
H37	0.39115	0.5198	0.6065
H38	0.34273	0.46954	0.65744
H39	0.30844	0.46173	0.61097
H40	0.27512	0.48789	0.6483
H41	0.3005	0.51513	0.59309
H42	0.36996	0.62878	0.15522
H43	0.35176	0.54904	0.33873
H44	0.50233	0.56931	0.41498
H45	0.45454	0.4371	0.52761
H46	0.67535	0.42809	0.35798
H47	0.696	0.45385	0.2997
H48	0.72307	0.41754	0.29451
H49	0.71327	0.39855	0.35854
H50	0.77916	0.4313	0.37889
H51	0.78981	0.45417	0.31707

H52	0.82758	0.42881	0.30076
H53	0.807	0.37981	0.29823
H54	0.81413	0.40155	0.35944
C55	0.33333	0.66667	0.30038
H56	0.66667	0.33333	0.84276

Section S2.4. Solid-state NMR spectra

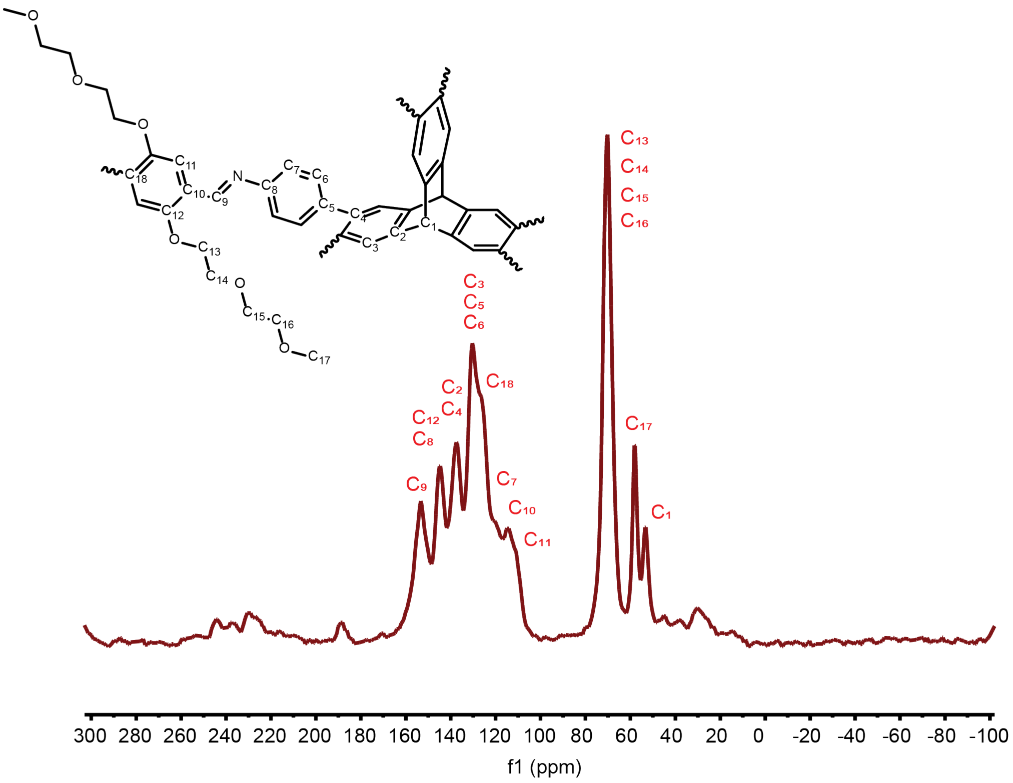

Figure S22. ^{13}C CP/MAS NMR spectra of HKU-2-PEG2.

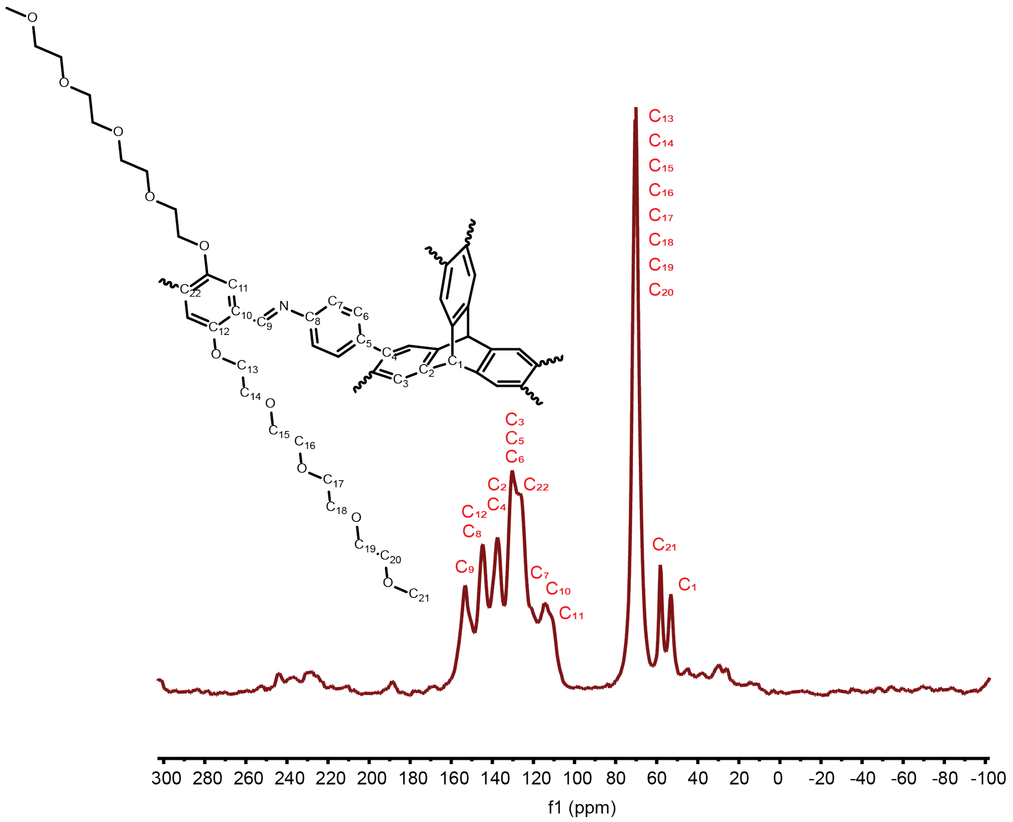

Figure S23. ^{13}C CP/MAS NMR spectra of HKU-2-PEG4.

Section S2.5. Fourier-transform infrared (FT-IR) spectra

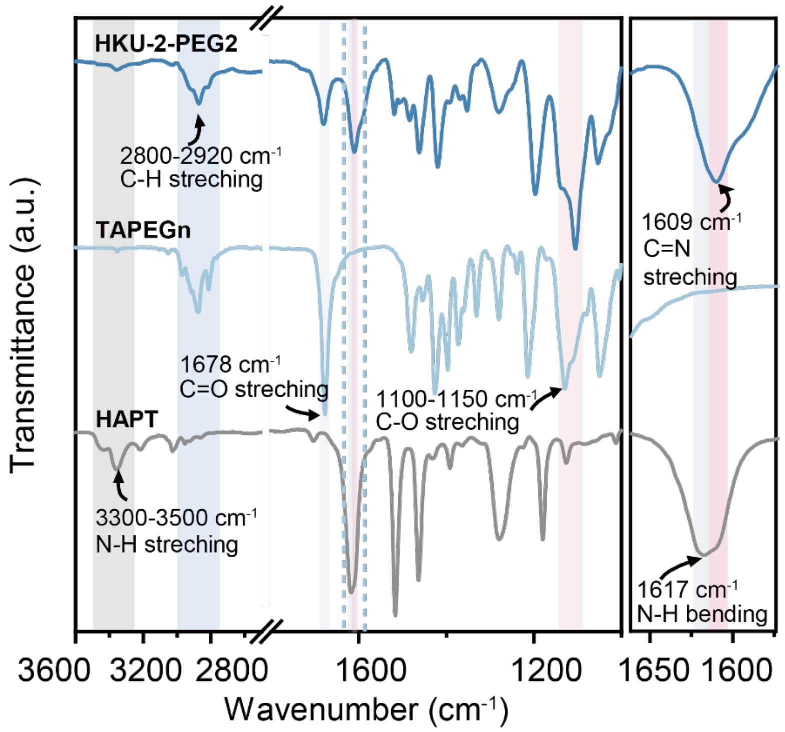

Figure S24. FT-IR spectra of HKU-2-PEG2.

Section S2.6. Channel characterization of HKU-2-PEGn

The channel widths reported in Figure 3 were determined directly from the graphical annotations. Specifically, the image scale was first calibrated using the 10 Å scale bar in Figure 3. The real-space separation between the two dashed lines marking the channel opening was then quantified, and this distance was taken as the effective pore aperture for the corresponding crystallographic direction. For each channel, the reported value corresponds to the dashed-line-to-dashed-line distance as shown in Figure 3. The flexible PEG side chains are treated as conformationally mobile and therefore are not included in the static geometric aperture definition.

Table S4. Multiplicity of Channel Directions in the Crystal Structure.

Representative channel direction	Number of symmetrically equivalent channels
[001]	1
[214]	6
[212]	6
[213]	6
[211]	6
[210]	3
[110]	3
[114]	6
[113]	6
[112]	6
[225]	6
[111]	6
[201]	6

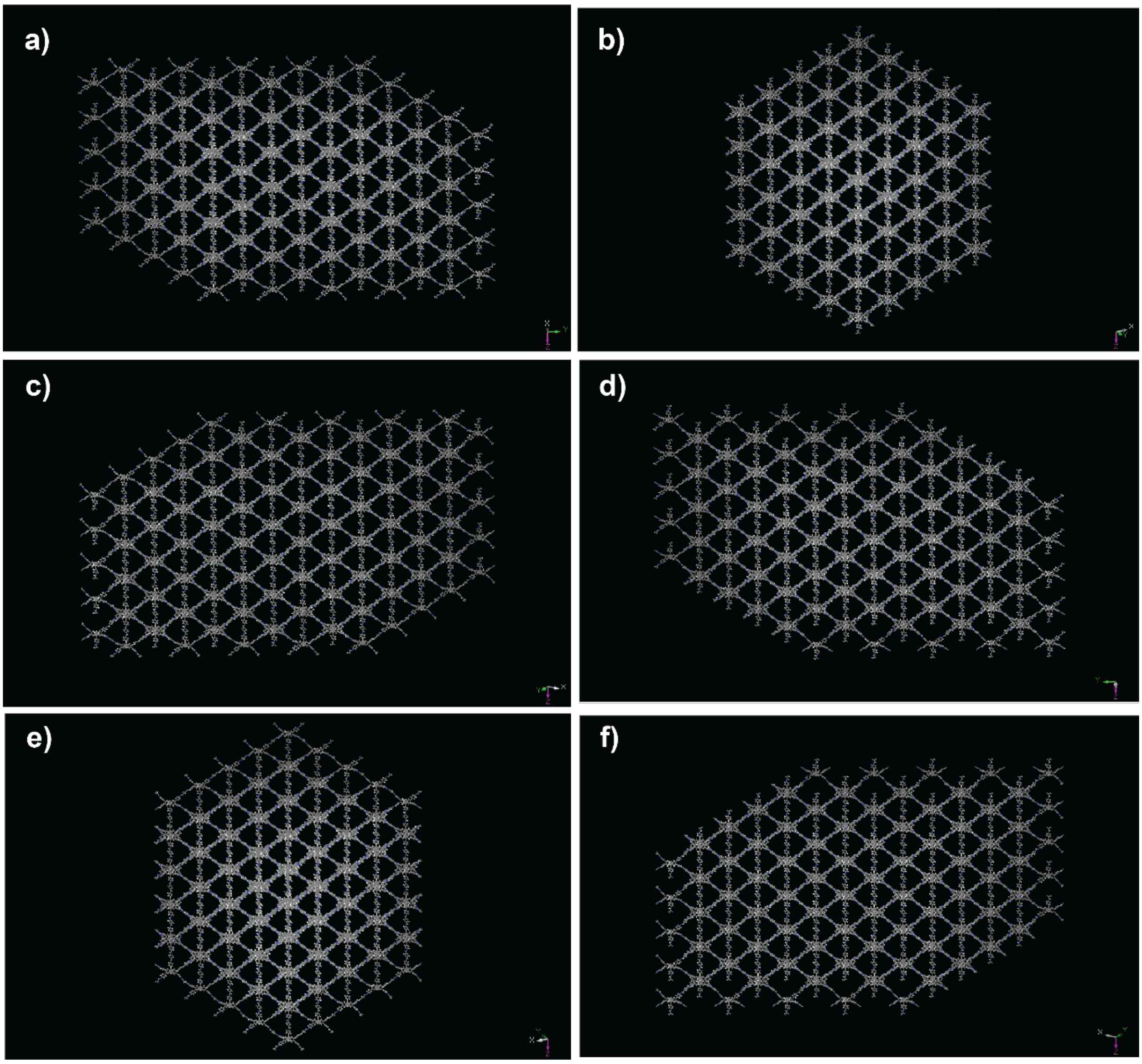

Figure S25. The complete set of six symmetry-equivalent channel directions for the [211] orientation.

Section S2.7. Optical microscopy images

Significant volume contraction was observed for HKU-2-PEGn upon solvent evaporation from the pores (Figure S26 and S27). We attempted to suppress this contraction—which likely induces pore collapse—by employing supercritical CO_2 drying; however, these efforts were unsuccessful. The pronounced contraction indicates that a substantial fraction of the pore

volume is occupied by solvent molecules, and that pore collapse occurs during the activation process.

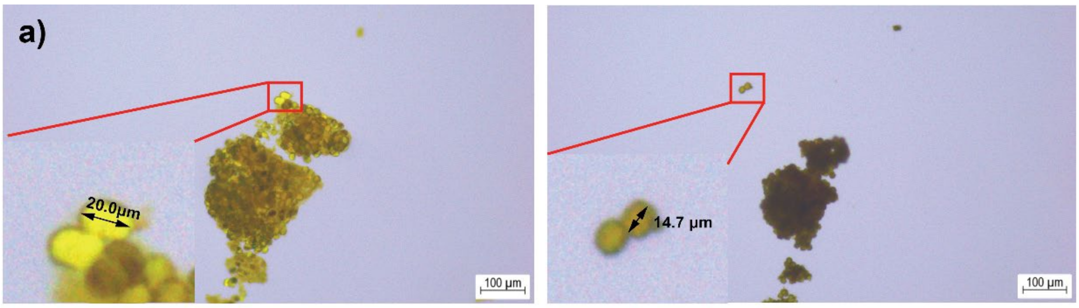

Figure S26. Optical microscopy images of HKU-2-PEG2 in (a) Ethanol and (b) air dry.

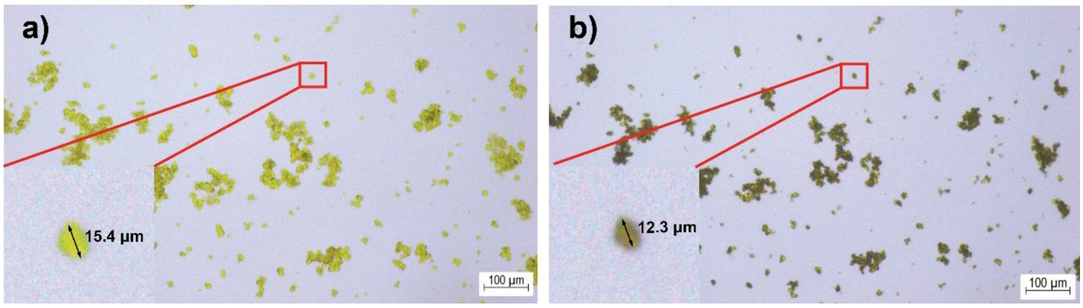

Figure S27. Optical microscopy images of HKU-2-PEG4 in (a) Ethanol and (b) air dry.

Section S2.8. N_2 isotherm measurements

The porosity of the COFs was studied by measuring nitrogen (N_2) isotherms at 77 K (Figure S28). The BET surface areas of HKU-2-PEG2 and HKU-2-PEG4 were 30 m^2/g and 3 m^2/g. The pore size distribution of HKU-2-PEG2 is 2.5 nm which are consistent with the pore size anticipated from the crystal structures according to the estimation of nonlocal density functional theory (NLDFT).

The low adsorption capacity of HKU-2-PEG4 likely arises from a combination of increased PEG chain length and the intrinsically more challenging activation behavior of framework with longer PEG chain.

First, compared with HKU-2-PEG2, the longer PEG chains in HKU-2-PEG4 occupy a larger fraction of the pore volume and partially reduce the accessible voids. Second, PEG-

functionalized COFs would exhibit stronger solvent retention and are generally more difficult to activate. Although supercritical CO_2 activation was employed, microscopy observations (Figures S26 and S27) suggest partial structural shrinkage during drying. These effects are consistent with the overall low BET surface areas observed for both samples (30 m^2/g for HKU-2-PEG2 and 3 m^2/g for HKU-2-PEG4).

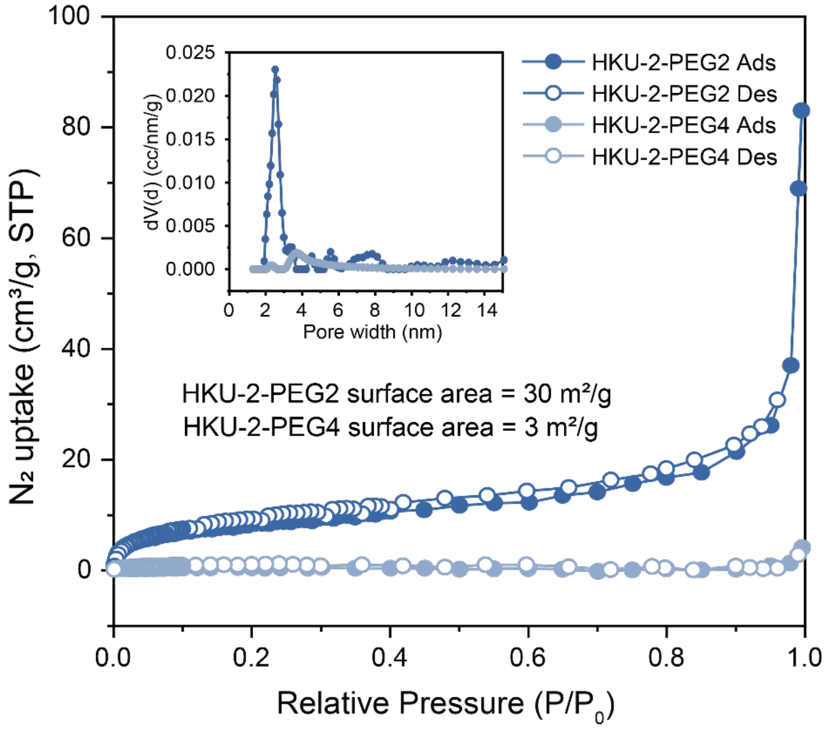

Figure S28. N_2 Isotherm of HKU-2-PEG2 and HKU-2-PEG4.

Section S2.9. Thermogravimetric analysis (TGA)

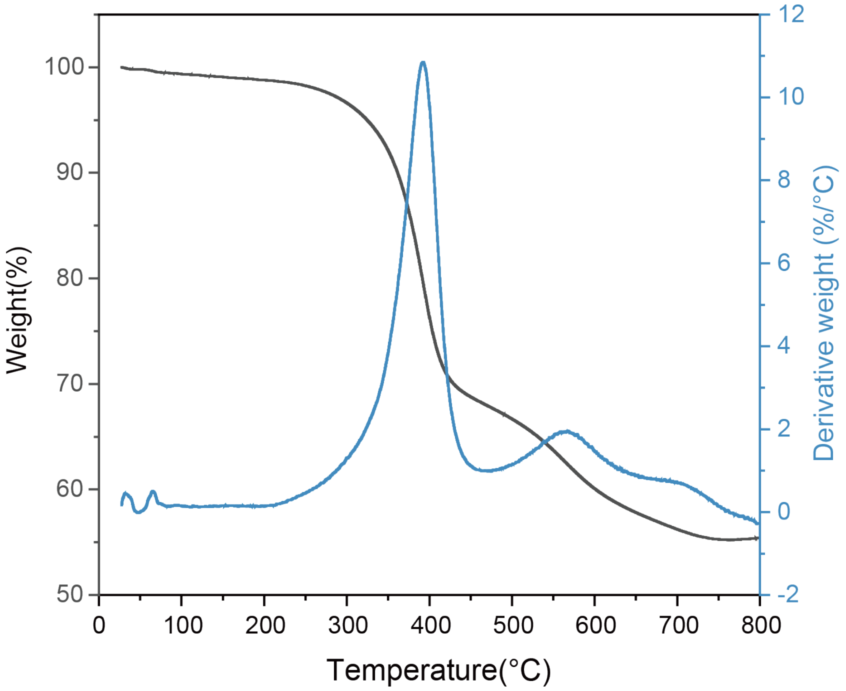

Figure S29. TGA of HKU-2-PEG2.

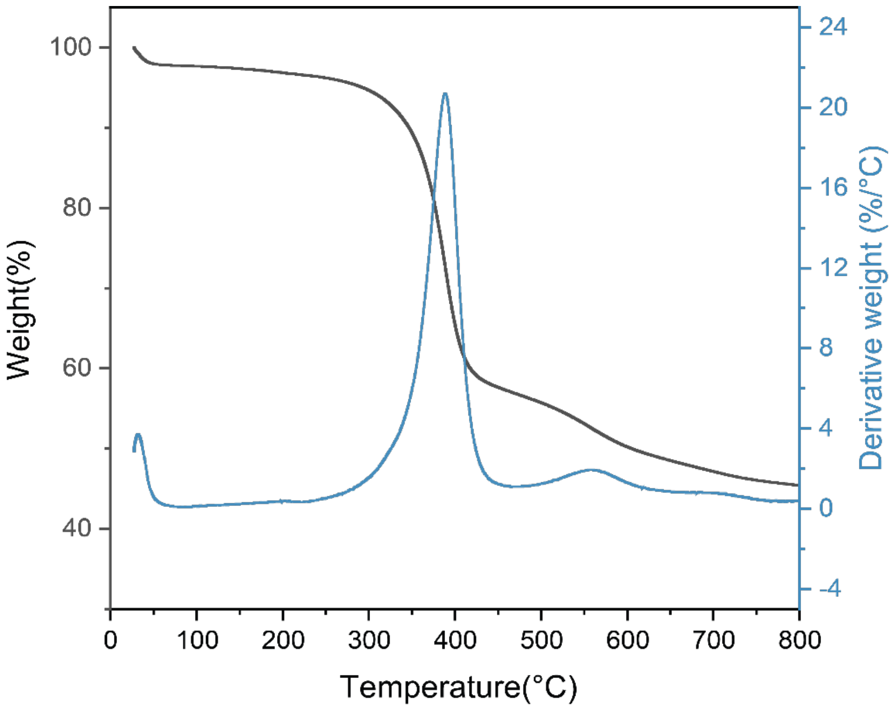

Figure S30. TGA of HKU-2-PEG4.

Section S2.10. Transmission electron microscopy (TEM) images

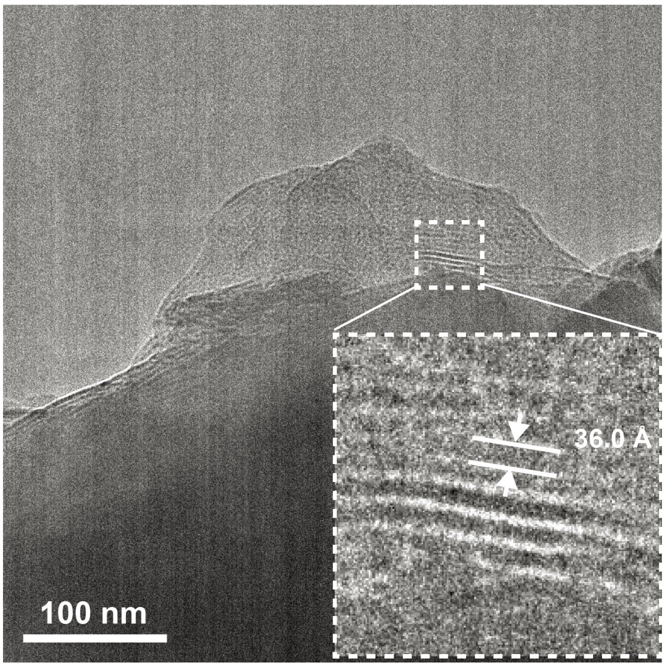

Figure S31. TEM image of HKU-2-PEG2.

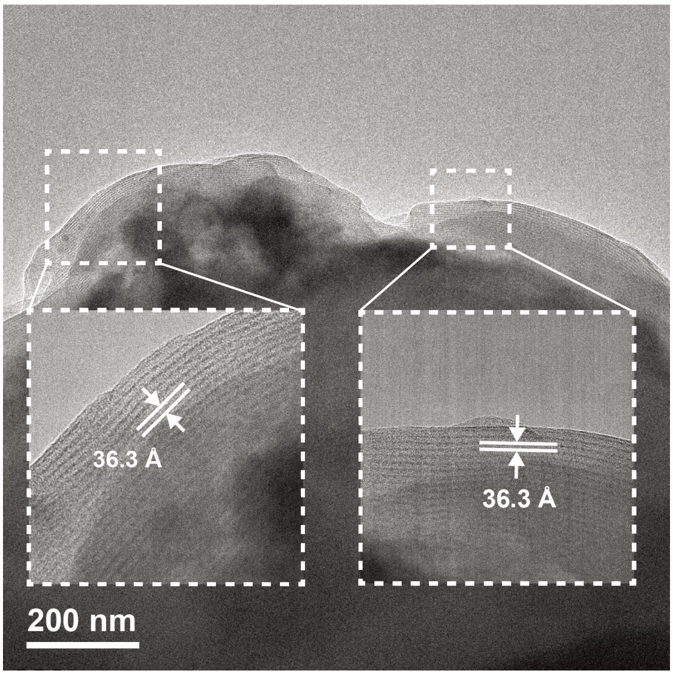

Figure S32. TEM image of HKU-2-PEG4.

Section S2.11. Stability test

To evaluate the chemical stability of the frameworks, HKU-2-PEG2 and HKU-2-PEG4 were immersed in water, 1 M HCl, and 6 M NaOH for 1 day.

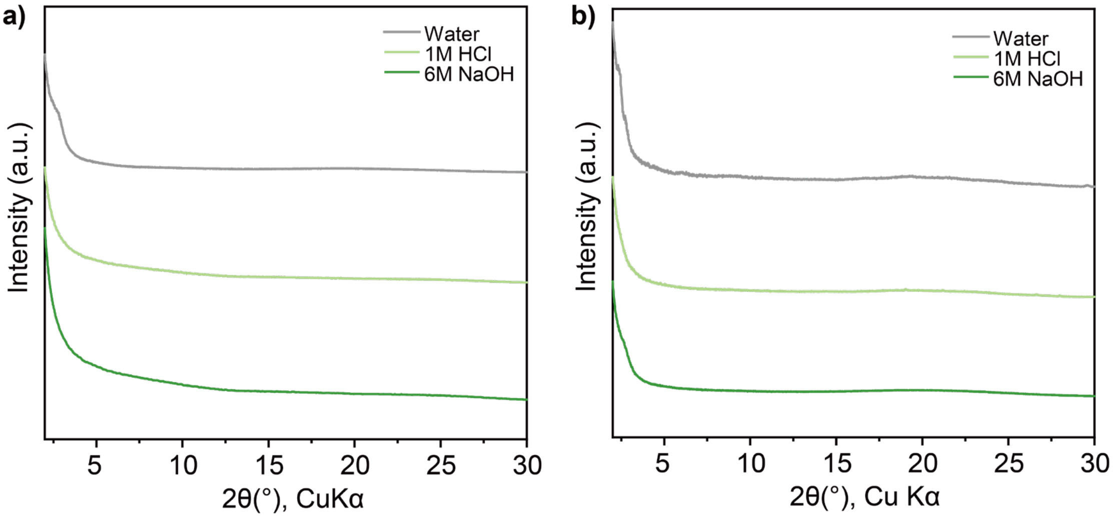

Figure S33. PXRD patterns of (a) HKU-2-PEG2 and (b) HKU-2-PEG4 after stability test.

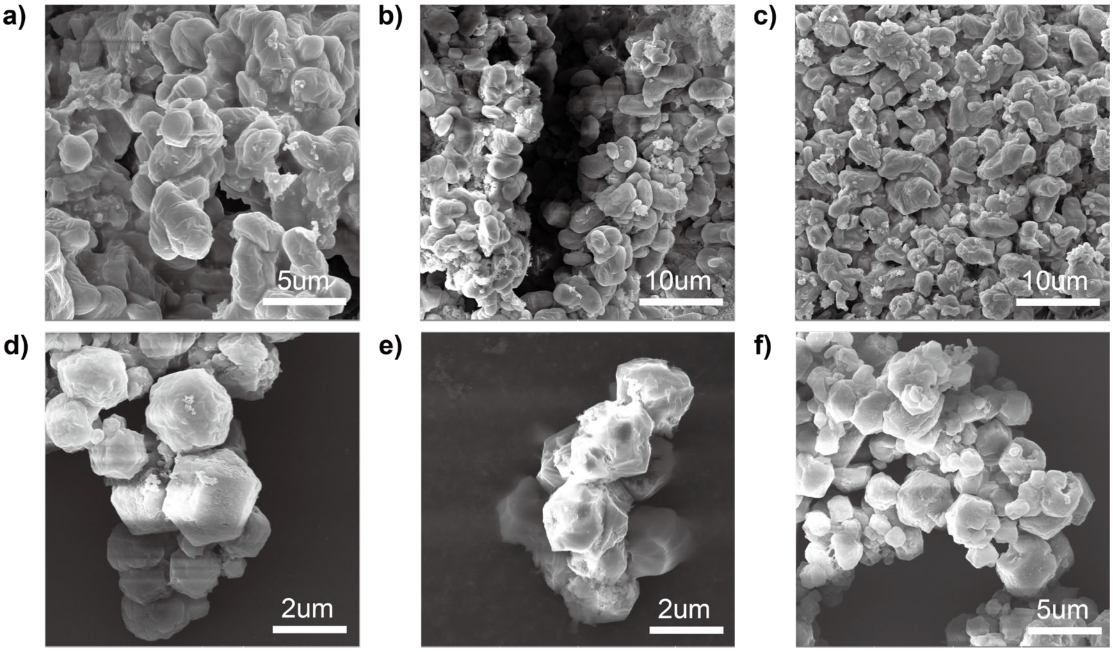

Figure S34. SEM images of HKU-2-PEG2 after treatment in (a) water, (b) 1 M HCl, (c) 6 M NaOH, and HKU-2-PEG4 after treatment in (d) water, (e) 1 M HCl, (f) 6 M NaOH.

Section S3. Conductivity studies

Section S3.1. Li^+ conductivity

Electrochemical impedance spectroscopy (EIS) ionic conductivities of all samples were determined by EIS (Amiral Squidstat Plus) over a frequency range of 0.1 Hz to 1 MHz with an AC potential of 10 mV. The solid electrolyte (SE) pellets were prepared by adding ~80 mg of the COFs powder to a stainless-steel die (6 mm). The as-prepared SE pellets were solvated by 40 wt.% PC. Then, each sample was sandwiched between stainless-steel electrodes in a Swagelok Cell during tests. Final ionic conductivity was calculated by following equation:

$$\sigma = \frac{L}{R * S}$$

Where L stands for thickness, R stands for bulk resistance of material, and S stands for area of tested sample.

Temperature dependent ionic conductivity was investigated. Swagelok Cell was heated from room temperature to 80 °C. And activation energy of the material was calculated according to Arrhenius equation:

$$\sigma = Aexp\frac{-E_a}{RT}$$

Where σ is ionic conductivity, A is pre-exponential factor, E_a is activation energy, R is ideal gas constant, T is temperature.

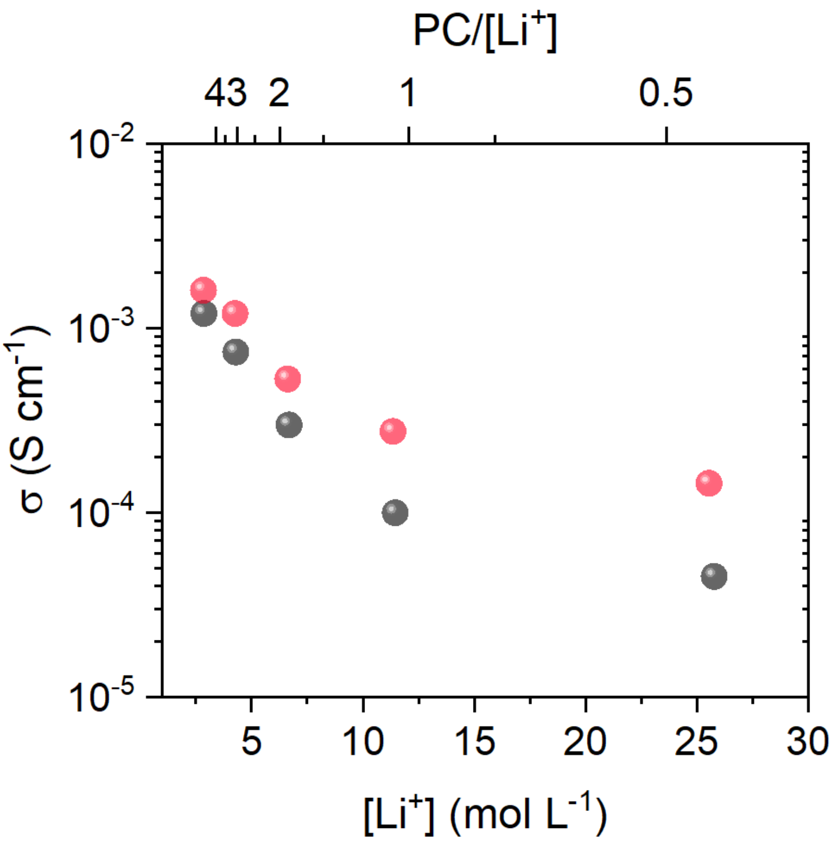

Figure S35. Ionic conductivity of Li^+@HKU-2-PEGn as a function of Li^+ concentration.

Section S3.2. Li^+ transference number

t_{Li+} is a crucial metric for electrolyte, high t_{Li+} indicate low concentration polarization during cycling. t_{Li+} was determined by chronoamperometry method and EIS. Lithium symmetric cells were prepared, and EIS was performed with a frequency range from 1MHz to 100MHz before and after polarization. Chronoamperometry was conducted with an applied potential of 10 mV. Gathered parameters were plugged into Bruce's equation:

$$t_+ = \frac{I^{ss}(\Delta V - I^0 R_{ct}^0)}{I^0(\Delta V - I^{ss} R_{ct}^{ss})}$$

Where ΔV is applied polarization DC voltage, I^0 and I^{ss} stands for initial and steady-state current before and after polarization, R_{ct}^0 and R_{ct}^{ss} stands for initial and steady-state charge transfer resistance before and after polarization.

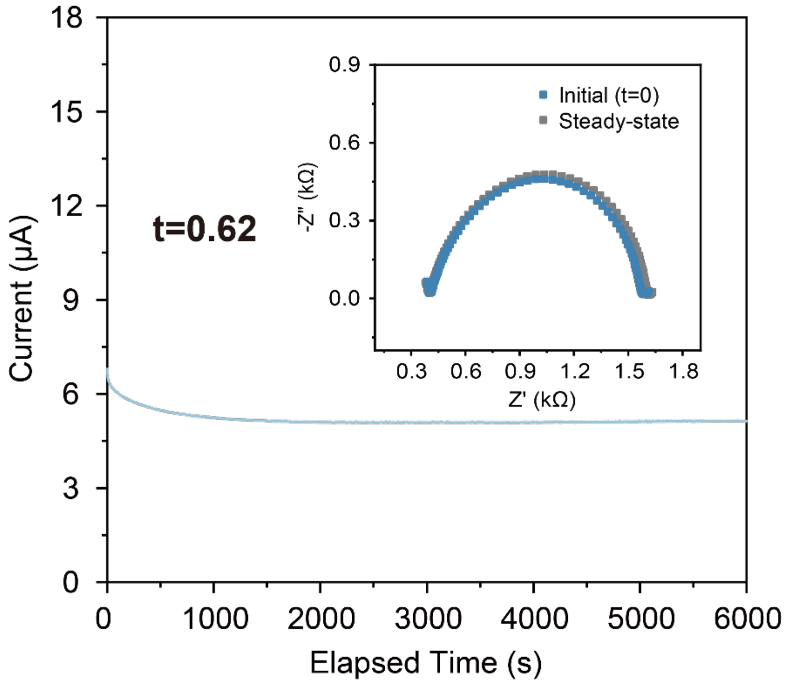

Figure S36. Electrochemical impedance spectra before and after polarization at 10 mV and the corresponding polarization curves for Li^+@HKU-2-PEG2 soaked in 4.3 M LiTFSI in PC solution.

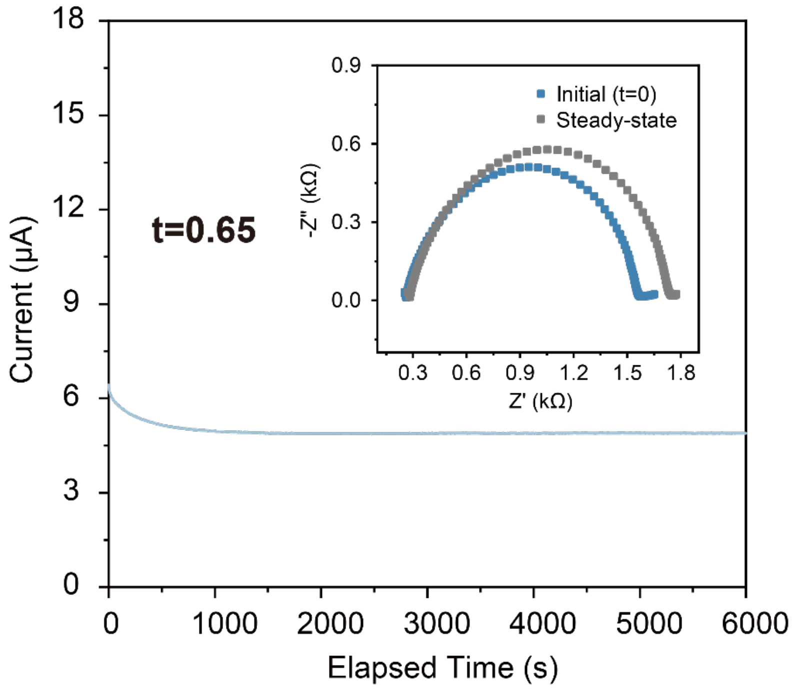

Figure S37. Electrochemical impedance spectra before and after polarization at 10 mV and the corresponding polarization curves for Li^+@HKU-2-PEG4 soaked in 4.3 M LiTFSI in PC solution.

Section S3.3. Electrochemical stability

Cyclic voltammetry (CV) was conducted to assess the electrochemical stability window and plate/stripping capability of the COF electrolyte. COF materials were packed between lithium plate and stainless-steel electrodes in coin cell. Stainless steel acted as working electrode and lithium plate acted as counter electrode and reference electrode. The cell was then left overnight to equilibrate the interface. To investigate the electrochemical stability window and reversibility, the voltage sweeps from -0.5 V to 4 V at 0.2 mV/s for 10 cycles.

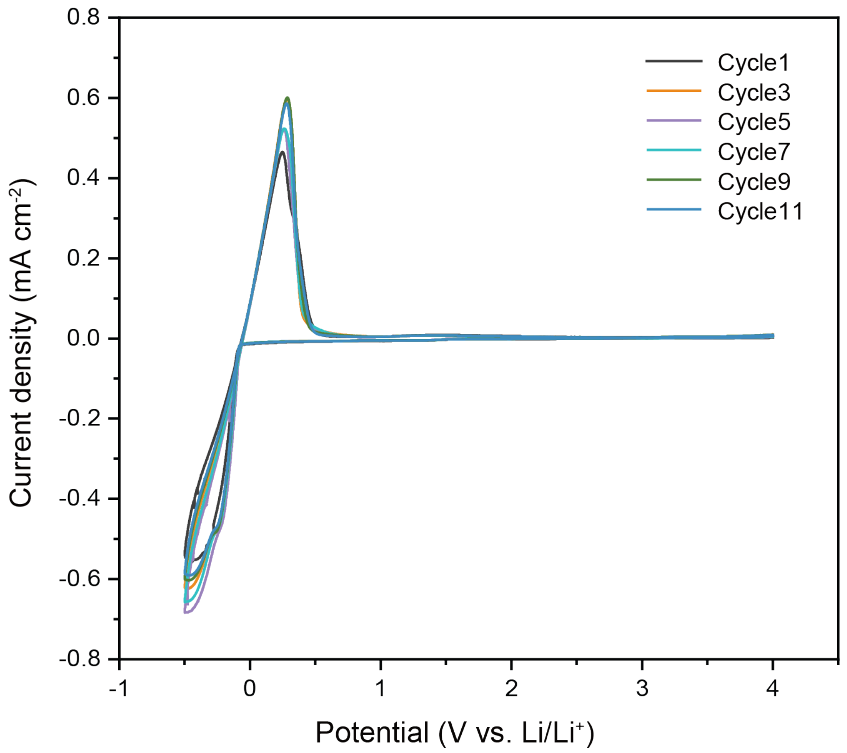

Figure S38. CV curve of Li^+@HKU-2-PEG2.

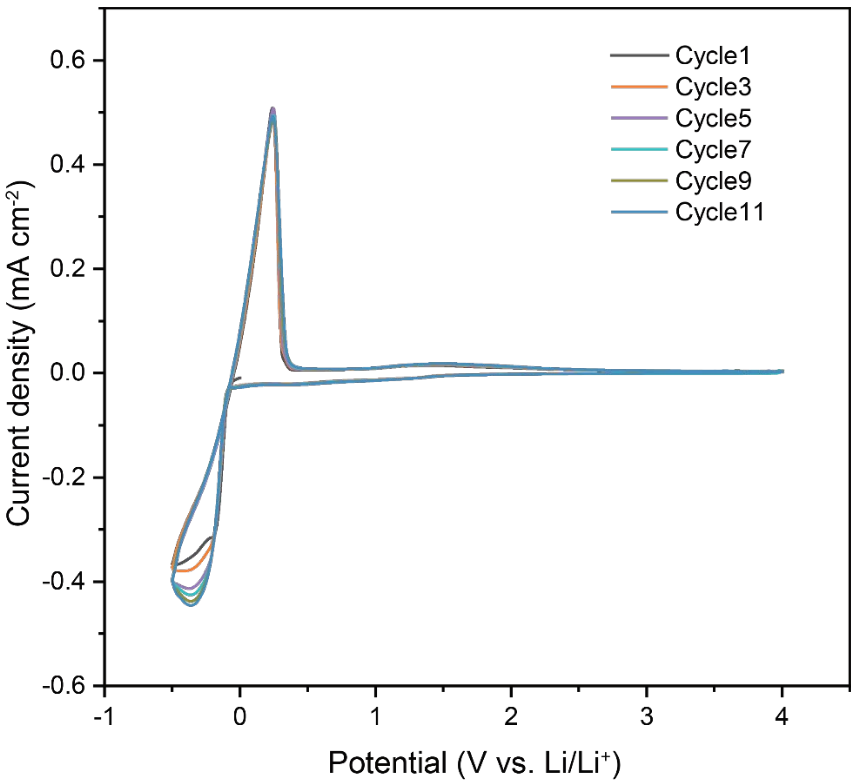

Figure S39. CV curve of Li^{+}@HKU-2-PEG4.

Section S3.4. Calculation of Diffusion Coefficient

Diffusion coefficient of lithium ions in electrolyte was calculated using Nernst-Einstein equation[3]:

$$\Lambda_m^0 = \frac{F^2}{RT} * (v_+ z_+^2 D_+ + v_- z_-^2 D_-)$$

where Λ_m^0 is limiting molar conductivity, which can be approximated by σ/C where C is Li^{+} concentration in the system. The lithium concentration was quantified via inductively coupled plasma optical emission spectrometry (ICP-MS, Agilent 7700), and the lithium concentrations of Li^{+}@HKU-2-PEG2 and Li^{+}@HKU-2-PEG4 were found to be 1.6545 wt.% and 1.6398 wt.%, respectively. F is the Faraday constant, R is the gas constant, T is the thermodynamic temperature, v_+ and v_- are the number of cations and anions per formula unit of electrolyte, z_+ and z_- are the valences of the ions, D_+ and D_- are the diffusion coefficients of the ions, t_+ is transference number of cations. From the definition of transference number ($t_+ = \frac{D_+}{D_+ + D_-}$), we can replace D_- with D_+ and then D_+ can be derived from the rearrangement. All parameter values are summarized in Table S5.

$$D_+ = \frac{\Lambda_m^0 RT}{F^2} / \left(v_+ z_+^2 + v_- z_-^2 \left(\frac{1 - t_+}{t_+} \right) \right)$$

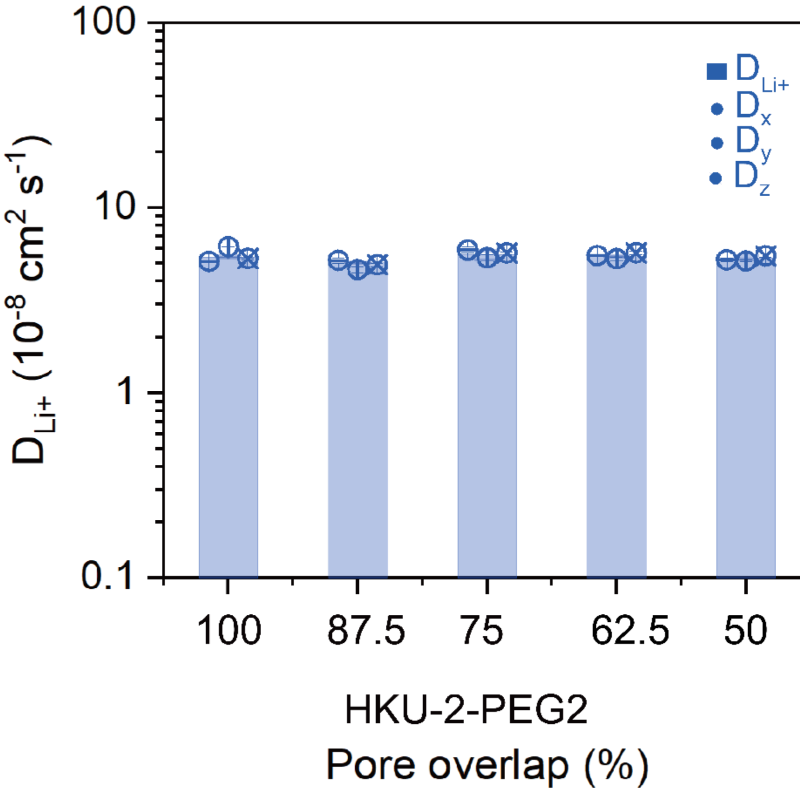

Figure S40. Diagonal diffusion coefficients for Li^+ in Li^+@HKU-2-PEG2 COF.

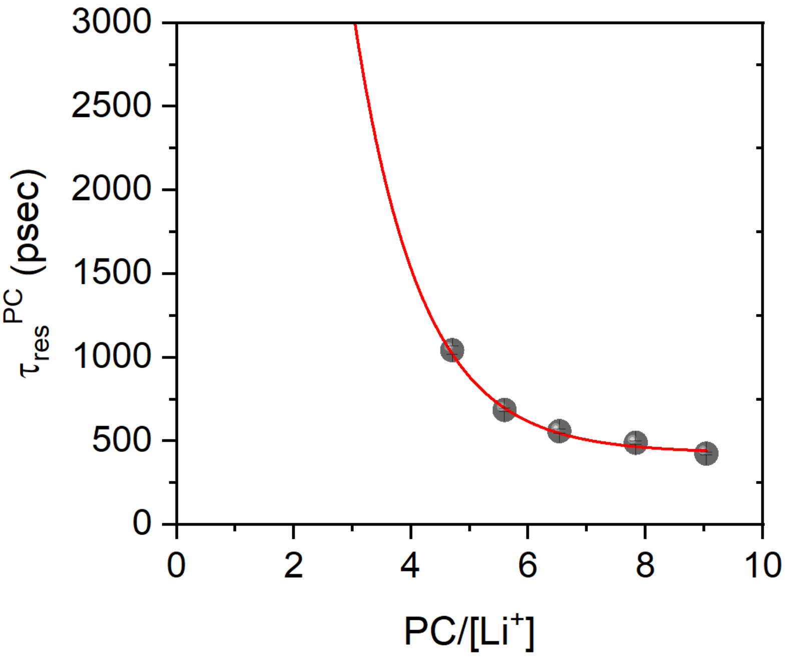

Figure S41. The residence time of a solvation-shell member (τ_{res}^{PC}) as a function of the ratio of PC to Li^+ (data taken from reference[2]). The red line is an extrapolation from five experimental data points.

Table S5. Ionic conductivity, Li^+ concentration in the system, molar conductivity, and molar ratio of Li^+ in COFs to the total PC added.

Samples	σ (S cm^{-1})	C (mol mL^{-1})	Λ_m^0	[Li^+] in PC (mol L^{-1})	PC/[Li^+]
HKU-2-PEG2					
10 wt% PC added	4.5×10^{-5}	4.34×10^{-3}	0.0104	25.7	2.7
20 wt% PC added	9.95×10^{-5}		0.0229	11.4	1.8
30 wt% PC added	2.98×10^{-4}		0.0686	6.7	1.0
40 wt% PC added	7.4×10^{-4}		0.1705	4.3	0.5
HKU-2-PEG4					
10 wt% PC added	1.44×10^{-4}	4.88×10^{-3}	0.0295	25.5	2.8
20 wt% PC added	2.75×10^{-4}		0.0564	11.3	1.8
30 wt% PC added	5.29×10^{-4}		0.1084	6.6	1.0
40 wt% PC added	1.20×10^{-3}		0.2452	4.3	0.5

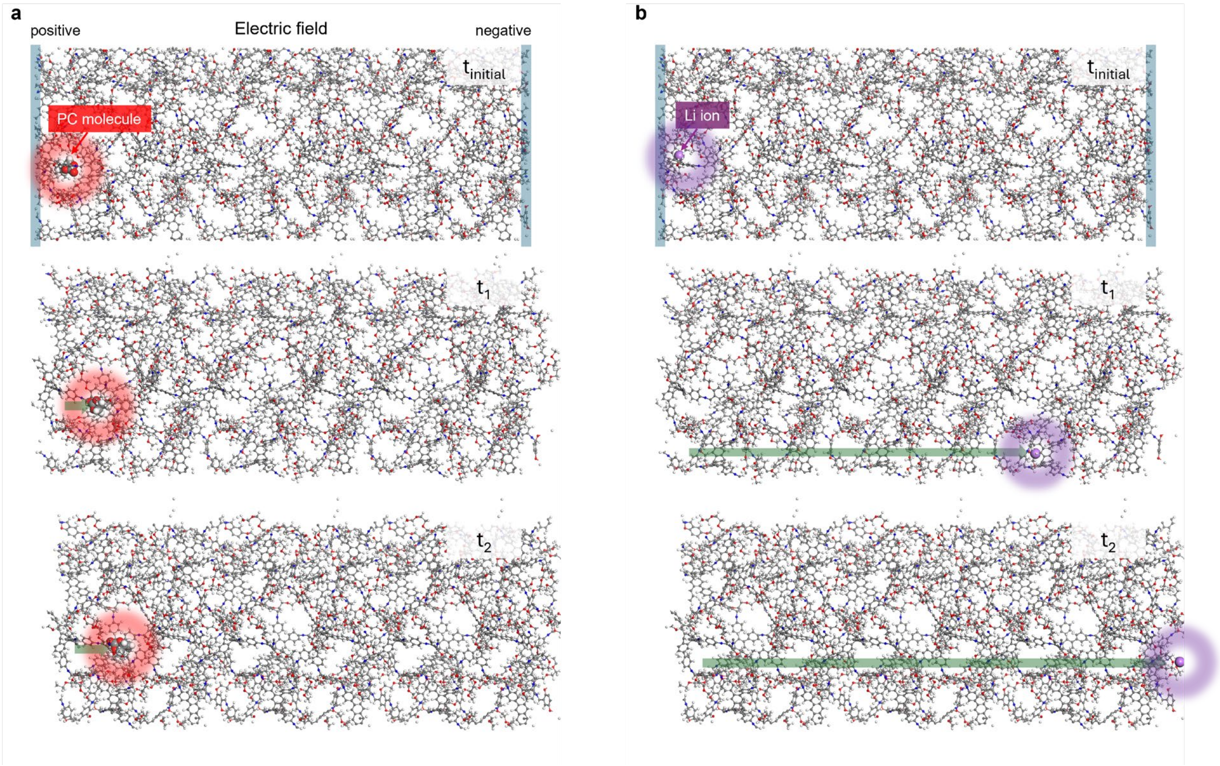

Figure S42. Snapshots of PC molecule (a) and Li^+ cation (b) transport pathways throughout HKU-2-PEG2.

Section S3.5. Molecular dynamic simulations

All calculations were carried out in Materials Studio 2024. The initial COF structures, HKU-2-PEG2 and HKU-2-PEG4, were first constructed using Materials Visualizer. Based on the experimental densities, PC molecules and LiTFSI were then introduced into the COF frameworks. The Mulliken charge distribution of each atom in the structures was calculated using the ORCA software. Temperature was controlled by application of Andersen thermostat. The simulations were carried out in the NVE, NVT, and NPT ensembles, respectively, with a time step of 0.5 fs. The temperature was controlled using an Andersen thermostat and Berendsen barostat. The systems were heated to 298 K for at least 2 ns each step for fully relaxation. Electrostatic interaction and Van der waals interaction adopted Ewald summation and Atom-based summation, respectively, with a cut-off distance of 9.5 Å. Simulations of Li^+ transport under applied the electric field of 0.6 V/Å along the z axis, and snapshots of the trajectory were recorded every 1 fs. The diffusion coefficient of Li^+ was determined from its mean square displacement (MSD) in the simulated system. The MSD was calculated using the following equation:

$$MSD(\Delta t) = \frac{1}{\tau - \Delta t}\int_0^{\tau-\Delta t} [r(t-\Delta t) - r(t)]^2 dt = \langle [r(t-\Delta t) - r(t)]^2 \rangle$$

where τ is the total simulation time and $r(t)$ is the position of the Li^+ at time t. According to the Einstein equation, the slope of this equation defines the Li^+ diffusion coefficient D:

$$D = \frac{1}{6} \lim_{\Delta t \to \infty} \frac{dMSD}{d\Delta t}$$

The factor 6 is due to the six possible directions of movement in three-dimensional space.

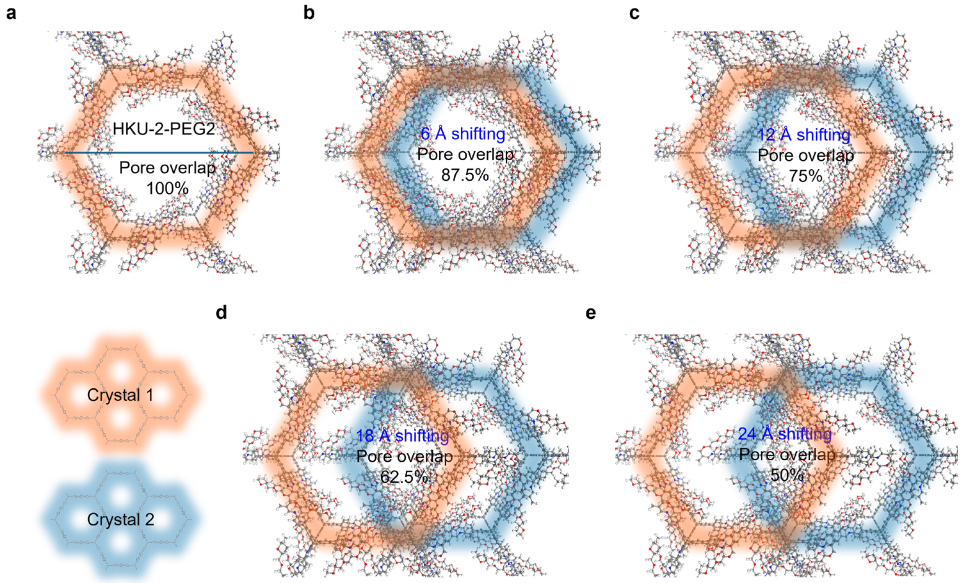

Figure S43. Schematic illustration of pore misalignment in Li^+@HKU-2-PEG2 with pore overlap range from 100% to 50%

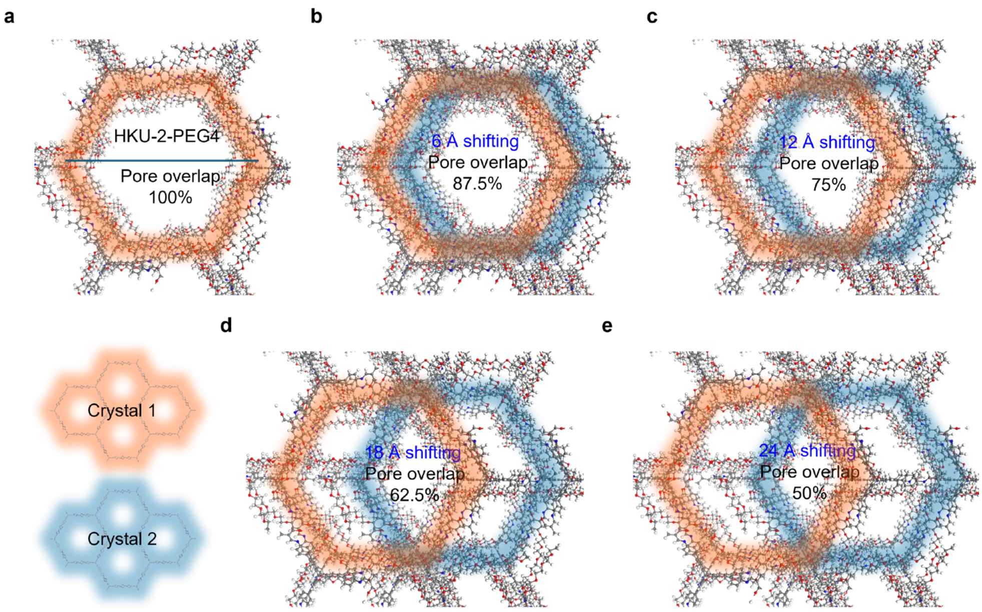

Figure S44. Schematic illustration of pore misalignment in Li^+@HKU-2-PEG4 with pore overlap range from 100% to 50%

Section S3.6. Characterization of Li^+@HKU-2-PEGn

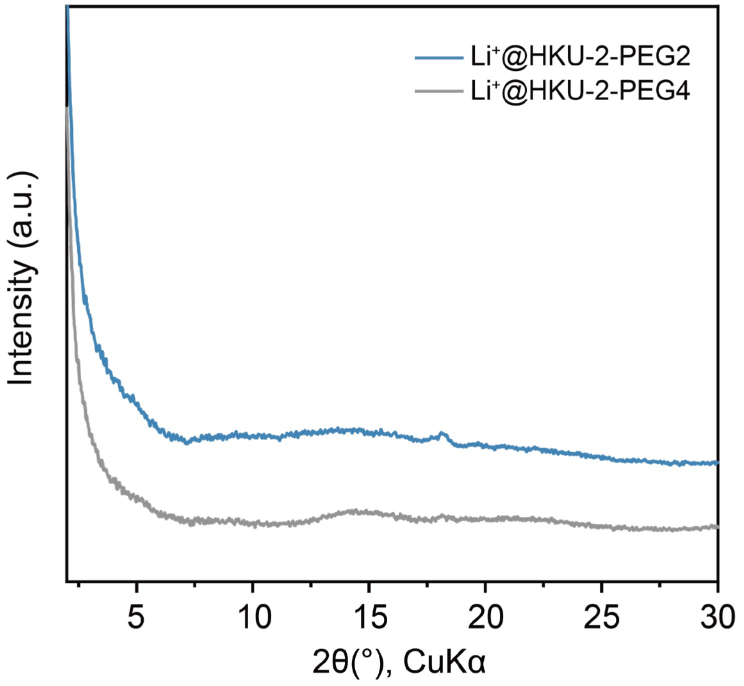

Figure S45. PXRD patterns of Li^+@HKU-2-PEG2 and Li^+@HKU-2-PEG4.

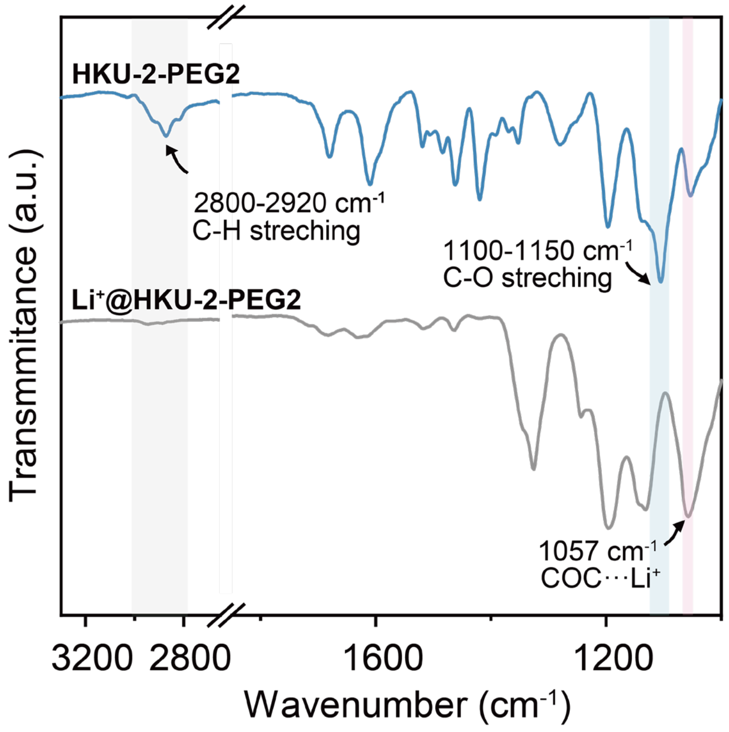

Figure S46. FT-IR spectra of Li^+@HKU-2-PEG2.

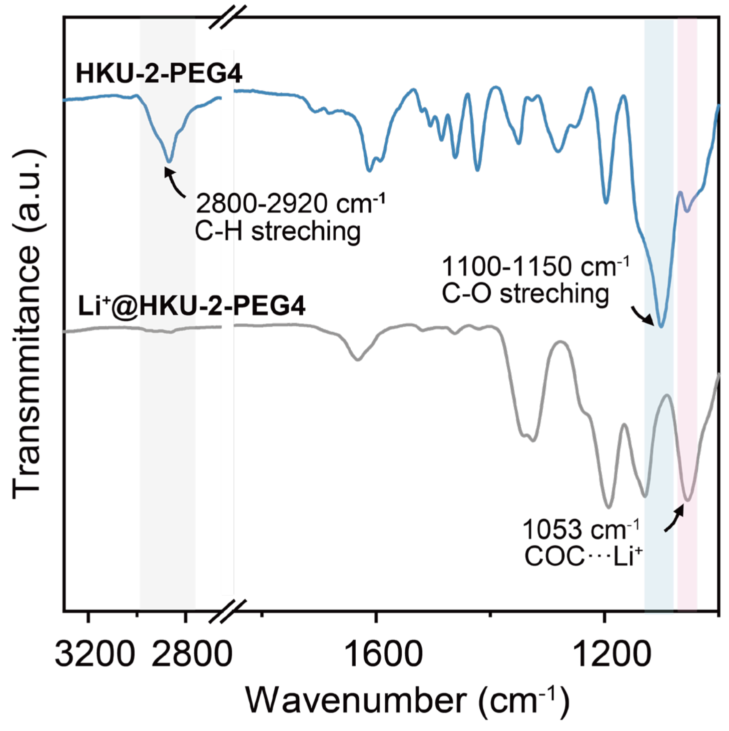

Figure S47. FT-IR spectra of Li^+@HKU-2-PEG4.

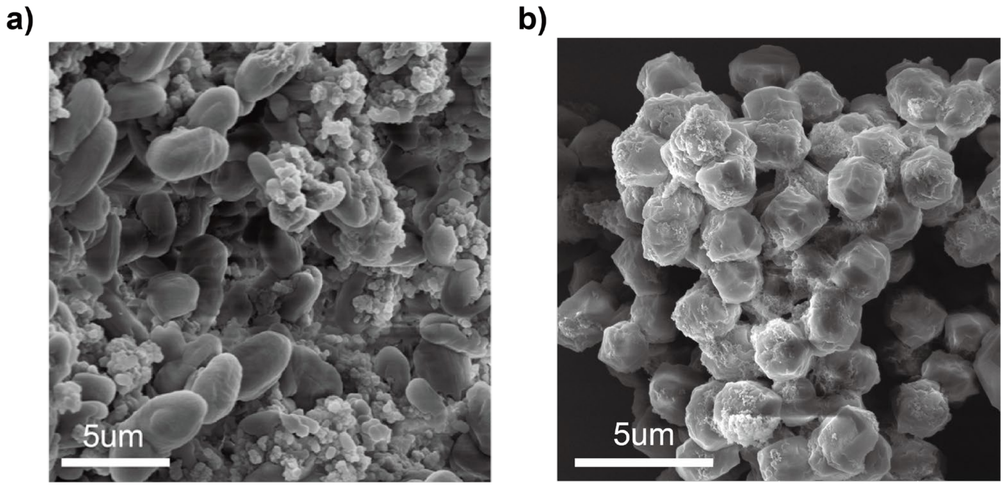

Figure S48. SEM images of (a) Li^+@HKU-2-PEG2 and (b) Li^+@HKU-2-PEG4.

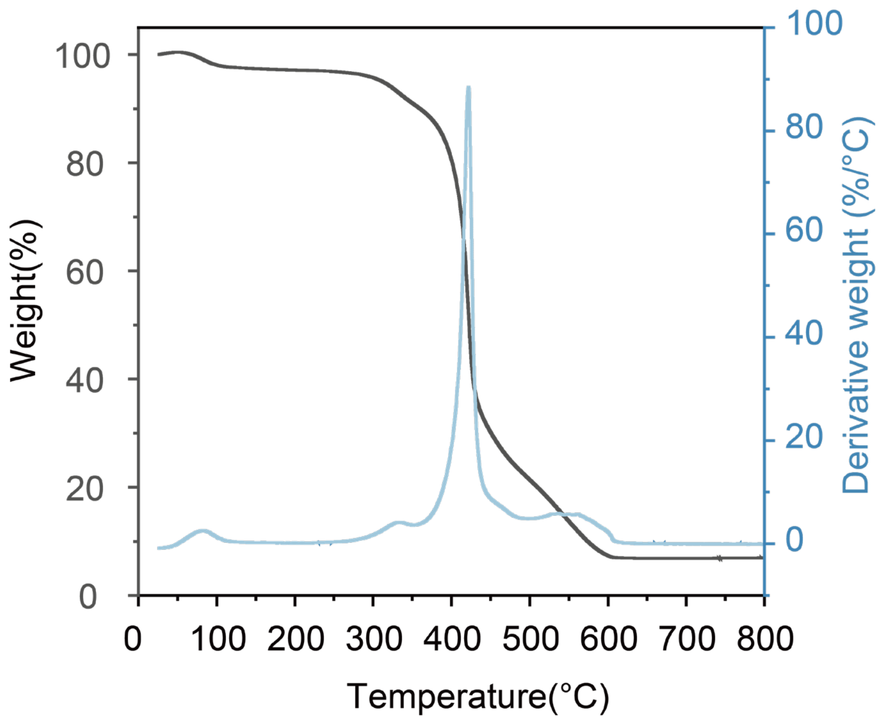

Figure S49. TGA of Li^+@HKU-2-PEG2.

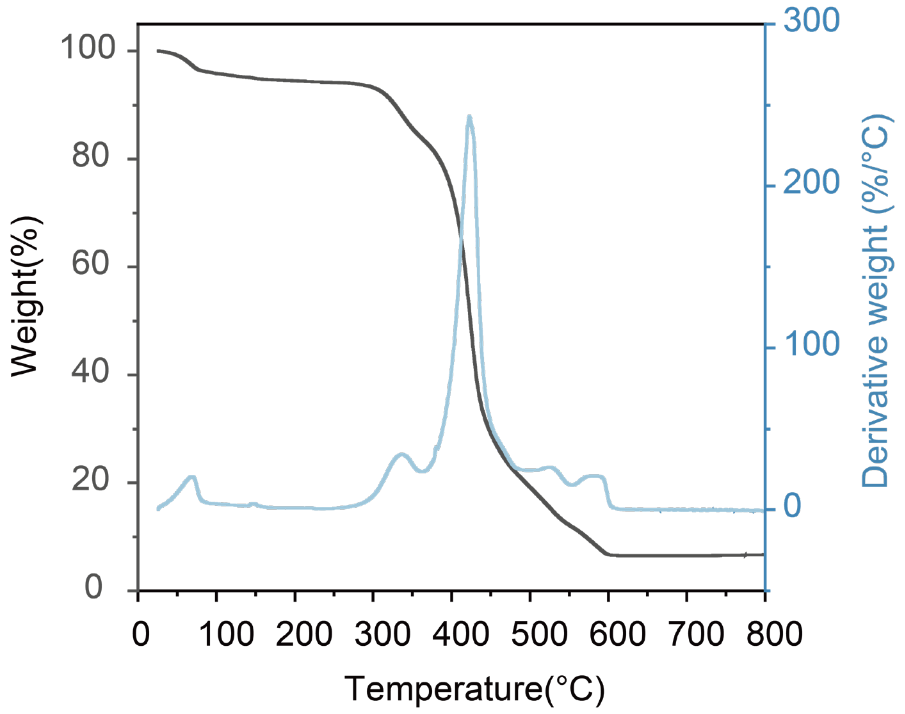

Figure S50. TGA of Li^+@HKU-2-PEG4.

References

(1) Norvez, S., Liquid crystalline triptycene derivatives. *J. Org. Chem.* **1993,** *58*, 2414-2418.
(2) Pan, J.; Charnay, A. P.; Zheng, W.; Fayer, M. D., Revealing Lithium Ion Transport Mechanisms and Solvation Structures in Carbonate Electrolytes. *J. Am. Chem. Soc.* **2024,** *146*, 35329-35338.
(3) Daintith, J., *A Dictionary of Chemistry*. Oxford University Press: 2008.
